# Non-invasive brain stimulation for stroke-related motor impairment and disability: an umbrella review of systematic review and meta-analysis

**DOI:** 10.3389/fnins.2025.1633986

**Published:** 2025-09-09

**Authors:** Beatriz Rithiely, Lívia Shirahige, Patrícia Lima, Maíra Souza, Déborah Marques, Rodrigo Brito, Adriana Baltar, Rafael J. Duarte-Moreira, Gabriel Barreto, Rodrigo Andrade, Kátia Nunes-Sá, Abrahão Fontes Baptista, Daniele Piscitelli, Kátia Monte-Silva

**Affiliations:** ^1^Universidade Federal de Pernambuco, Recife, Pernambuco, Brazil; ^2^Applied Neuroscience Institute, Recife, Pernambuco, Brazil; ^3^NAPeN Network (Núcleo de Assistência e Pesquisa em Neuromodulação), Recife, Brazil; ^4^Universidade Federal do ABC, São Bernardo do Campo, SP, Brazil; ^5^Universidade Federal do Ceará, Fortaleza, Ceará, Brazil; ^6^Escola Bahiana de Medicina e Saúde Pública, Salvador, Bahia, Brazil; ^7^Universidade Federal do Rio de Janeiro, Rio de Janeiro, RJ, Brazil; ^8^Department of Kinesiology, University of Connecticut, Storrs, CT, United States; ^9^School of Medicine and Surgery, University of Milano Bicocca, Milan, Italy

**Keywords:** stroke, transcranial magnetic stimulation, transcranial direct current stimulation, motor function, neurological rehabilitation, recovery, neuroplasticity, evidence-based practice

## Abstract

**Introduction:**

Non-invasive brain stimulation (NIBS) techniques, particularly repetitive transcranial magnetic stimulation (rTMS) and transcranial direct current stimulation (tDCS), have shown potential in stroke rehabilitation. However, systematic reviews often reach conflicting conclusions, highlighting the need for an umbrella review.

**Objective:**

To synthesize, based on the principal domains of the International Classification of Functioning, Disability and Health (ICF) framework, the best available evidence on the effectiveness and safety of NIBS for improving motor impairment and disability after stroke.

**Methods:**

We conducted an umbrella review (PROSPERO: CRD42021239577) that included meta-analyses of controlled trials investigating NIBS effects in stroke survivors, retrieved from PubMed/MEDLINE from February 2020 to July 2025. Methodological quality was appraised using AMSTAR-2 and certainty of evidence using GRADE. Outcomes were mapped to ICF body structure/function and activity domains.

**Results:**

Fifty-six studies were included (2–48 primary trials each; 54–1,654 participants per meta-analysis). All included studies evaluated only rTMS and tDCS; no meta-analyses of other NIBS modalities met inclusion criteria. Methodological quality was high or moderate in 85.7% of the meta-analyses. Certainty of evidence was low or very low for 14/50 studies; only one rTMS review provided moderate-certainty evidence for activities of daily living. rTMS showed improvement in activities of daily living (ADL; SMD = −0.82, 95% CI −1.05 to −0.59), upper-limb motor impairment (SMD = −0.32, 95% CI −0.55 to −0.09) and variable effects on mobility from small (SMD = −0.35, 95% CI −0.45 to −0.24) to large (SMD = −0.97, 95% CI −1.28 to −0.66). tDCS was supported by very-low-certainty evidence: small effects were found for motor impairment (SMD = −0.22, 95 % CI −0.32 to −0.12) and upper-limb activity (SMD = −0.31, 95% CI −0.55 to −0.01), while a much smaller subset of trials suggested a large effect (SMD = −1.54, 95% CI −2.78 to −0.29). Effects on ADL and mobility with tDCS were inconsistent and generally non-significant.

**Conclusion:**

rTMS was more frequently associated with moderate to large effect sizes for body structure/function outcomes, particularly general neurological function. In contrast, tDCS demonstrated small effects on motor recovery, though evidence certainty was very low due to heterogeneity, imprecision, and protocol variability. Within the activity domain, NIBS showed modest effects, with rTMS showing more consistent benefits for ADL. tDCS effects were generally limited and supported by low to very low certainty of evidence.

**Systematic review registration:**

https://www.crd.york.ac.uk/PROSPERO/view/CRD42021239577.

## 1 Introduction

Stroke is a leading cause of motor impairment and disability worldwide, consistently exerting a significant impact on public health across many countries (Virani et al., 2021). Non-invasive brain stimulation (NIBS) is a set of techniques that apply non-invasively electromagnetically-induced currents to modulate the excitability of the targeted brain areas and their networks (Brunoni et al., 2019). NIBS approaches might enhance or drive adaptive plastic changes in the central nervous system (CNS) for the management of various stroke-related sensorimotor impairments and disabilities, including spasticity (Graef et al., 2016; McIntyre et al., 2018), upper or lower motor function (Zhang et al., 2017a; Kang et al., 2018; Vaz et al., 2019), balance impairments (Li et al., 2018a; Ghayour-Najafabadi et al., 2019; Kang et al., 2020; Tien et al., 2020), mobility (Ghayour-Najafabadi et al., 2019; Tien et al., 2020) and difficulties with activities of daily living (Subramanian and Prasanna, 2018; Xiang et al., 2019; Ahmed et al., 2022).

In recent decades, NIBS has been proposed as a possible adjuvant strategy to augment the efficacy of conventional rehabilitation treatments for sensorimotor impairments in neurological populations (Liew et al., 2014). In the context of stroke rehabilitation, several NIBS modalities have been investigated (Kim and Park, 2024; Shen et al., 2022). However, repetitive transcranial magnetic stimulation (rTMS) and transcranial direct current stimulation (tDCS) have been the most extensively studied (Mahmoud et al., 2024). Although the quality of available evidence remains limited, numerous clinical studies suggest that NIBS holds promise for enhancing motor recovery after stroke. Recently, several systematic reviews have synthesized the growing body of evidence on NIBS (Qi et al., 2024; Barreto et al., 2025). However, the large number of available reviews may lead to conflicting conclusions and hinder consensus on the effectiveness of NIBS. To address this challenge, umbrella reviews have become increasingly important, providing a qualitative meta-synthesis of systematic reviews or meta-analyses. By synthesizing evidence across multiple reviews, umbrella reviews can help resolve inconsistencies and provide a comprehensive overview of findings. Thus, they are considered one of the highest levels of evidence synthesis currently available and have been used to inform the adoption of specific clinical techniques in practice (Aromataris et al., 2015; Liu et al., 2020).

Another important limitation of most existing reviews on NIBS for post-stroke motor recovery is their predominant focus on isolated clinical outcomes (e.g., motor scores or spasticity), without contextualizing the findings within a comprehensive functional framework that reflects real-world functioning. The International Classification of Functioning, Disability and Health (ICF) provides a comprehensive framework to address this gap by categorizing the consequences of stroke-related neurological damage across three core domains: impairments in body structures and functions, limitations in activity, and restrictions in participation (Leonardi and Fheodoroff, 2021). The ICF has become a standard for understanding and categorizing the multidimensional impact of health conditions such as stroke (Virani et al., 2021; The Lancet, 2019; Stucki et al., 2007).

In this context, we conducted an umbrella review to summarize the evidence on the use of NIBS techniques for motor recovery and disability reduction in stroke survivors, framing the synthesis within the core domains of the ICF. We conceptualize motor recovery as a multidimensional process that encompasses improvements in body structures and functions, as well as gains in activity performance. This umbrella review aims to enhance the clinical relevance of the synthesized findings and support more holistic interpretations of NIBS effects.

## 2 Materials and methods

### 2.1 Study design

This review is part of a series of umbrella reviews produced by the Working-Group on scientific evidence for the use of non-invasive brain stimulation within the NIBS Brazilian Guidelines Development Group of the NAPeN Network (http://www.neuromodulation-net.com). The protocol was registered in PROSPERO (CRD42021239577; February/2020) and subsequently published by Shirahige et al. (2022), following the recommendations of the preferred reporting items for overviews of reviews (PRIOR) statement (Gates et al., 2022).

### 2.2 Search and eligibility criteria

Two independent reviewers (BR and PL) conducted a comprehensive literature search from April 2020 to July 2025 in PubMed/MEDLINE. Disagreements during the screening process were resolved through discussion to reach a consensus; if consensus could not be achieved, a third reviewer (LS) was consulted. The search strategy was developed and validated in consultation with specialists in scientific methodology and experts in NIBS. These experts reviewed the selection of keywords, controlled vocabulary terms, and Boolean operators to ensure the adequacy, sensitivity, and specificity of the search process in line with the aims of this umbrella review.

To enhance the comprehensiveness of the meta-analysis, the snowball method was also employed, identifying additional relevant studies through reference lists and forward citation tracking, thereby ensuring comprehensive inclusion of pertinent literature. We included meta-analyses of controlled trials (CTs) involving any NIBS technique used as a treatment for motor impairments and disability in stroke survivors. Searches were conducted using Medical Subject Headings (MeSH) terms. The NIBS techniques included were: transcranial direct current stimulation (tDCS), transcranial alternating current stimulation (tACS), transcranial random noise stimulation (tRNS), transcutaneous spinal direct current stimulation (tsDCS), transcutaneous auricular vagus nerve stimulation (taVNS), high-definition transcranial direct current stimulation (HD-tDCS), and repetitive transcranial magnetic stimulation (rTMS). The scope of the search was guided by the list of electrical and magnetic NIBS modalities most frequently reported in the scientific literature, as outlined by experts from the NAPeN Network Group. Eligibility criteria are summarized in Box 1. All strategies including respective MeSH terms and number of retrieved articles are described in Supplementary Table 1.

**Box 1:** Eligibility criteria for considering articles for the umbrella review.

**Criteria**	**Inclusion**	**Exclusion**
Population (P)	Adult subjects with stroke who have been treated with one of the NIBS techniques	Animal studies
Intervention (I)	tDCS, rTMS, tACS, tRNS, tsDCS, taVNS, and tsMS	Association of two or more active NIBS techniques in the same intervention
Comparison (C)	Sham NIBS or no intervention associated or not with another approach of treatment (i.e., physical therapy, occupational therapy, cognitive training, etc.)	Comparison between two active NIBS techniques (ex. rTMS vs. tDCS)
Outcome (O)	Changes in outcome measurements	Surrogate outcomes
Study design (S)	Systematic reviews with meta-analysis of CT randomized or not; published in English	Meta-analysis without qualitative analysis Meta-analysis published before 2015 Network meta-analyses Studies from which it was not possible to extract or convert the data into SMD
rTMS, cerebellar repetitive transcranial magnetic stimulation; CT, clinical trials; HD-tDCS, high-definition transcranial direct current stimulation; NIBS, non-invasive		
brain stimulation; RCT, randomized clinical trials; rTMS, repetitive transcranial magnetic stimulation; tACS, transcranial alternating current stimulation; TBS, theta		
burst stimulation; tcDCS, transcranial cerebellar direct current stimulation; tDCS, transcranial direct current stimulation; tRNS, transcranial random noise stimulation;		
tsDCS, transcutaneous spinal direct current stimulation; taVNS, transcutaneous auricular vagus nerve stimulation.		

### 2.3 Study selection and data extraction

Titles, abstracts and full texts were screened by two independent reviewers (BR and PL) to assess study eligibility. Disagreements during screening were resolved through discussion to reach consensus; if consensus could not be reached, a third reviewer (LS) was consulted. The following data were extracted from each included study: (1) author/year of publication; (2) characteristics of patients from selected articles; (3) intervention protocols used in the articles; (4) number of patients, number of patients included in the meta-analysis, heterogeneity index, and *p*-value; (5) adverse effects: tissue damage, behavioral changes and vasovagal syncope; (6) outcome measures used in each meta-analysis.

Severe adverse events included incapacitating headaches, seizures, syncope, psychiatric and cognitive/neuropsychological changes, and tissue injury. Results of each meta-analysis were extracted separately for each outcome. All data were checked to ensure accuracy and consistency in two steps. Any discrepancies were resolved by consensus. Outcome measures were classified according to the principal domains of the ICF conceptual framework, body structure/function and activity, based on the approach proposed by Salter et al. (2019). In addition, we specified all outcome measures used in the included studies within the corresponding ICF subdomains, as detailed in Supplementary Table 2.

### 2.4 Assessment of meta-analyses methodological quality

The quality of the included systematic reviews was assessed using the AMSTAR-2 (A Measurement Tool to Assess Systematic Reviews) checklist (Shea et al., 2017). This instrument evaluates 16 domains that evaluate methodological quality. Specifically, the following aspects were considered: inclusion of PICO components in the research question and eligibility criteria (Item 1); prospective registration of the review protocol and justification for deviations (Item 2); justification for the selection of study designs (Item 3); use of a comprehensive literature search strategy (Item 4); study selection performed in duplicate (Item 5); data extraction performed in duplicate (Item 6); listing and justification of excluded studies (Item 7); adequate description of included studies (Item 8); appropriate assessment of risk of bias in individual studies (Item 9); reporting of funding sources for the included studies (Item 10); use of appropriate methods for statistical combination of results (Item 11); consideration of risk of bias when interpreting results of the meta-analysis (Item 12); consideration of risk of bias in the discussion or interpretation of the review findings (Item 13); explanation of heterogeneity in the review results (Item 14); assessment of publication bias and its potential impact on findings (Item 15); and disclosure of conflicts of interest and funding for the review itself (Item 16).

Each item was rated as “Yes,” “Partially yes,” or “No,” with emphasis placed on seven critical items that significantly impact the overall score (Shea et al., 2017). The quality of each included meta-analysis was assessed by considering non-critical items (1, 3, 5, 6, 8, 10, 12, 14, and 16) and critical items (2, 4, 7, 9, 11, 13, and 15).

Based on ratings for critical and non-critical items, the systematic reviews were categorized into one of four quality levels: “high quality” (no or one non-critical weakness), “moderate quality” (more than one non-critical weakness), “low quality” (one critical flaw with or without non-critical weaknesses), and “critically low” (more than one critical flaw with or without non-critical weaknesses; Shea et al., 2017). Methodological quality assessments were performed independently by two researchers. Any disagreements were resolved through discussion, and if consensus was not reached, a third reviewer was consulted.

### 2.5 Assessment of evidence quality

Data were extracted into Summary of Finding tables using GRADEpro GDT (Grading of Recommendations Assessment, Development and Evaluation Guideline Development Tool; http://www.gradepro.org). Data was organized according to the main domains of the ICF. Separate tables were created for each NIBS technique addressing outcomes of ICF body structure/function and activity. The GRADE approach provides a quality rating for each outcome as high, moderate, low, or very low. *High-quality evidence* indicates that future studies are unlikely to change the effect size estimate; *moderate-quality evidence* suggests that future RCTs may have an impact on the effect size estimate; *low-quality evidence* indicates that there is a high probability that future studies will change the effect size estimate; and *very low-quality evidence* indicates that there is very little certainty about the effect size estimate.

### 2.6 Statistical analysis

Given the considerable variability in the NIBS protocols across studies and the use of different instruments to assess body structure/function, we used the standardized mean difference (SMD) as the treatment effect for continuous outcome measures. This approach allowed for the standardization of results across studies. Pooled SMDs were calculated as the overall treatment effect size in the meta-analyses (Gallardo-Gómez et al., 2024). We interpreted pooled SMDs using rules of thumb (< 0.40 = small, 0.40–0.70 = moderate, >0.70 = large effect) according to the *Cochrane Handbook for Systematic Reviews of Interventions* (Higgins et al., 2024).

When original meta-analyses reported outcomes only as mean differences, we re-analyzed the post-intervention data by extracting the mean and standard deviation (SD) from each included study and generated new forest plots using SMDs. If means and SDs were not provided, median values were considered to be equal to mean values if data were normally distributed, and interquartile ranges were divided by 1.35 to obtain the SD (Higgins and Welch, n.d.). When necessary, we also derived the SD from confidence intervals, following the Cochrane Handbook (Higgins and Welch, n.d.). When the study only presented the results in graphs, we extracted the data using WebPlotDigitizer (available at https://apps.automeris.io/wpd/). All adjusted meta-analyses were performed using RevMan 5 software (Cochrane Information Management System).

To enhance the clinical interpretability of the effect sizes reported in our systematic review, we converted standardized mean differences (SMDs) into approximate estimates of the Number Needed to Treat (NNT), following the approach proposed by Furukawa and Leucht (2011). The conversion was performed using the following formula:


$$\begin{array}{l} \text{NNT}=\frac{1}{\text{Φ}\left(\frac{\text{SMD}}{\sqrt{2}}\right)}-0.5 \end{array}$$

Where Φ is the cumulative distribution function (CDF) of the standard normal distribution, and SMD is the standardized mean difference for the outcome of interest. This approach allows for a rough but informative approximation of NNT from continuous outcomes. The resulting NNT values, along with their corresponding 95% confidence interval (NNT lower and higher), were added to a Supplementary Table 2 alongside the original SMD, to support clinical interpretation. Negative SMDs, where applicable, were interpreted in the context of the direction of benefit, and the sign was adjusted accordingly when calculating NNT.

All statistical tests were two-sided, with significance set at p ≤ 0.05. Homogeneity was evaluated by a heterogeneity test. A meta-analysis was considered homogeneous when the *p*-value was >0.05 and the heterogeneity index (*I*^2^) was up to 30%. When heterogeneity was >30%, a random-effects model was used, whereas a fixed-effect model was used when *I*^2^ was ≤ 30%. The Supplementary Table 2 provides the specific measure considered for each main domain of the ICF included in the meta-analyses.

## 3 Results

### 3.1 Study selection and characteristics of included meta-analyses

**Figure 1:**
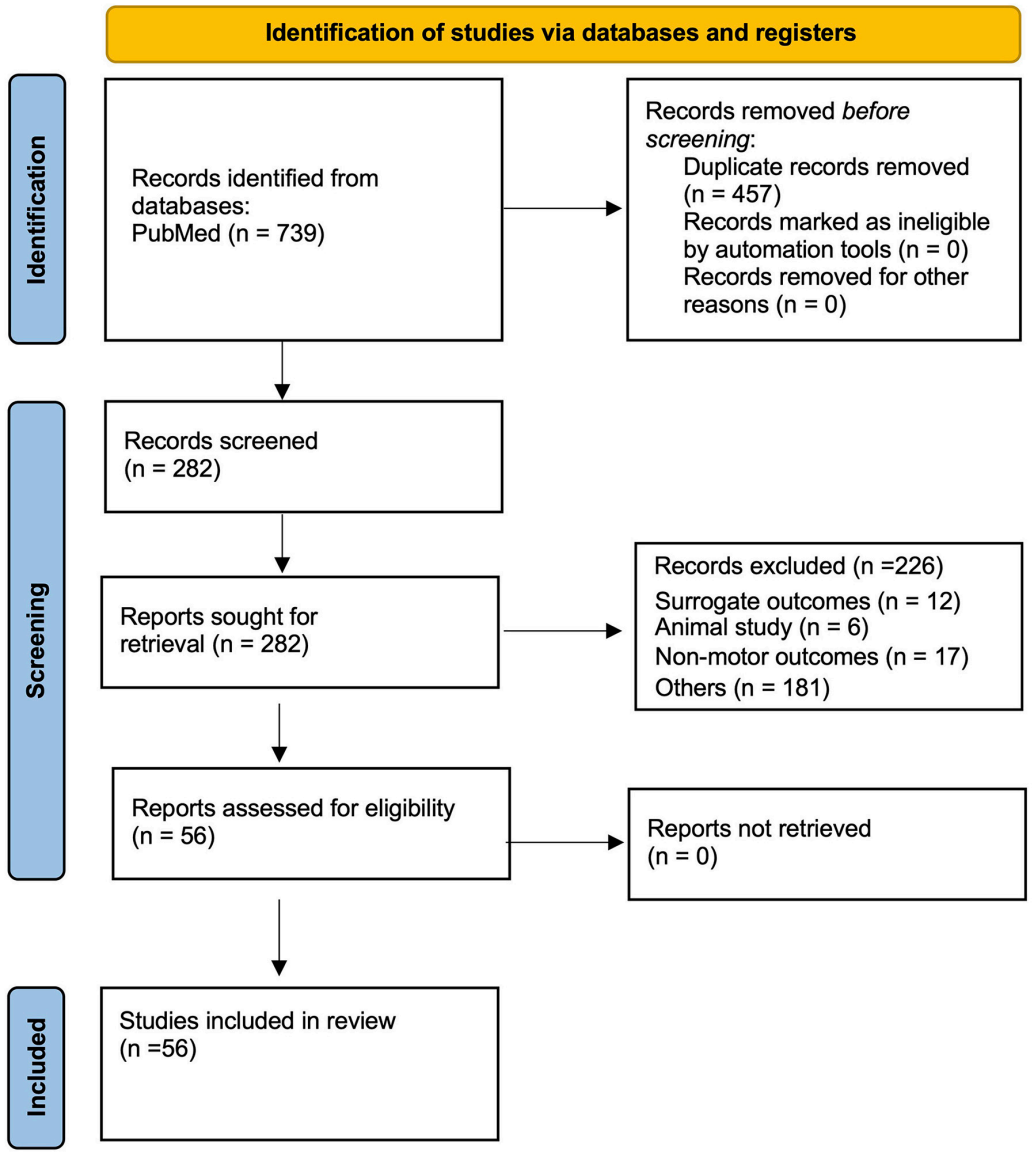
PRISMA flowchart.

A total of 56 systematic reviews with meta-analysis met the eligibility criteria and were included in the study. All retrieved studies focused exclusively on the efficacy of rTMS and tDCS, with no eligible meta-analyses found for other NIBS modalities. The screening strategy is shown in the Preferred Reporting Items for Systematic Reviews and Meta-analyses (PRISMA) flowchart presented in Figure 1.

The included studies were published between 2016 (Elsner et al., 2016) and 2025 (Barreto et al., 2025). Of the 56 studies included, 35 evaluated the efficacy of rTMS (Graef et al., 2016; Shen et al., 2017; Wang et al., 2025; Zhang et al., 2017a,b; Li et al., 2018a; McIntyre et al., 2018; Ghayour-Najafabadi et al., 2019; Liu et al., 2019; Tung et al., 2019; van Lieshout et al., 2019; Xiang et al., 2019; Allida et al., 2020; Shao et al., 2021; Krogh et al., 2022; Gao et al., 2023; Hofmeijer et al., 2023; Chen X. et al., 2023; Chen Y. et al., 2023; Xie et al., 2023, 2025; Xi et al., 2023; Zhou et al., 2023; Chen et al., 2024; Daoud et al., 2024; Jiang et al., 2024; Zhang J. J. et al., 2024; Wang J. et al., 2024; Wang Y. et al., 2024; Zeng et al., 2024; Zhu et al., 2024; Barreto et al., 2025; Jia et al., 2025; Ma et al., 2025; Zhang et al., 2025) whereas 16 evaluated that of tDCS (Elsner et al., 2016, 2020; Tedesco Triccas et al., 2016; Li et al., 2018b; Tien et al., 2020; Van Hoornweder et al., 2021; Comino-Suárez et al., 2021; Reis et al., 2021; Sun et al., 2021; Huang et al., 2022; Lima et al., 2023, 2024; Zhang N. et al., 2024; Tang et al., 2024; Usman et al., 2024; Yu et al., 2025). Notably, 5 meta-analyses evaluated both rTMS and tDCS within the same review (O'Brien et al., 2018; Vaz et al., 2019; Kang et al., 2020; Ahmed et al., 2023; Ren et al., 2024). The number of primary studies included in each meta-analysis ranged from 2 (McIntyre et al., 2018; Allida et al., 2020; Ahmed et al., 2023) to 48 (Zhou et al., 2023), and the number of participants ranged from 54 (Xi et al., 2023) to 1654 (Xie et al., 2025).

Control interventions included sham stimulation, or no intervention associated with physiotherapy, occupational therapy, task-oriented training, mirror therapy, treadmill training, usual care, constraint-induced movement therapy, or pharmacological interventions. The characteristics of the included meta-analyses are summarized in Table 1.

**Table 1:** Main characteristics of the included meta-analyses.

**References**	**Outcomes**	***I*^2^/ heterogeneity *p*-value**	**Adverse events**	**Stimulation target**	**Stimulation protocol**	**Number of sessions**	**Measures**	**Comparison group**
Elsner et al. (2016)	Motor function—tDCS	82%; < 0.01	Not reported	atDCS (affected M1) or ctDCS (unaffected) or Bi-tDCS	I: 0.5–2 mA; D: 13–20 min; ES: 18–35 cm^2^	15–30	MAS	Sham tDCS or Sham tDCS + virtual reality or physical therapy
Graef et al. (2016)	Motor function—TMS	0%; 0.44	No	M1 (unaffected side)	F: 1 Hz; T: 1; P: 240–1,800; MT (%): 90	10–22	FMA-UL	Sham rTMS + repetitive facilitation exercises or CIMT or physical therapy
Graef et al. (2016)	Upper limb activity—TMS	52%; 0.02	No	M1 (unaffected or affected side)	F: 1–20 Hz; T: 1–50; P: 1,200–2,000; MT (%): 90–110	8–22	WMFT	Sham rTMS + task-oriented training or physical therapy or CMIT or occupational therapy
Tedesco Triccas et al. (2016)	Motor function—tDCS	0%; 0.99	Yes (headache and dizziness)	atDCS (affected M1), ctDCS (unaffected M1) or bihemispheric M1	I: 1–2 mA; D: 13–40 min; ES: NR	5–30	FMA-UL	Sham tDCS + physical therapy or occupational therapy or CIMT or virtual reality
Tedesco Triccas et al. (2016)	ADL—tDCS	33%; 0.20	Yes (headache and dizziness)	atDCS (affected M1), ctDCS (unaffected M1) or bihemispheric M1	I: 2 mA; D: 20–25 min; ES: NR	15–30	BI	Sham tDCS + occupational therapy or CIMT or virtual reality
Shen et al. (2017)	General neurological function—TMS	53%; 0.05	Yes (Headache, gastrointestinal reaction, tinnitus and feel weak)	lDLPFC or rDLPFC or M1 or bilateral DLPFC	F: 0.5–10 Hz; T: 30; P: 1,500; MT (%): 60–110	7–24	NIHSS	Regular treatment or Sham rTMS + regular treatment or antidepressant
Shen et al. (2017)	ADL—TMS	89%; < 0.01	Yes (Headache, gastrointestinal reaction, tinnitus and feel weak)	lDLPFC or rDLPFC or M1 or bilateral DLPFC	F: 0.5–10 Hz; T: 20–30; P: NR; MT (%): 60–100	10–60	BI	Sham rTMS + antidepressant or fluoxetine or sertraline or mirtazapine or regular treatment
Zhang et al. 2017a)	Motor function—TMS	52%; 0.04	Not reported	M1 (unaffected side)	F: 1 Hz; T: NR; P: 600–1,800; MT (%): 80–130	10–24	FMA-UL; Pinch force; Hand grip	Sham rTMS + physical therapy or occupational therapy or functional task practice or task-oriented training
Zhang et al. 2017a)	Upper limb activity—TMS	0%; 0.46	Not reported	M1 (unaffected side)	F: 1 Hz; T: NR; P: 600–1,800; MT (%): 90–100	1–24	JTT; NHPT; WMFT	Sham rTMS + rehabilitation or task-oriented training or functional task practice or occupational therapy or extensor activity
Li et al. 2018a)	Motor function—TMS	0%; 0.72	Not reported	M1 (unaffected or affected side) of the leg area	F: 1–20 Hz; T: 1–30; P: 600–2,000; MT (%): 90	1–40	FMA-LL	Sham rTMS or Sham rTMS + task-oriented training
Li et al. 2018a)	Mobility—TMS	0%; 0.53	Not reported	M1 (unaffected or affected side) of the leg area	F: 1–20 Hz; T: 1–30; P: 600–2,000; MT (%): 90	1–40	TUG; 10 MWT; Gait analysis	Sham or Sham + MI + rehab or Sham + rehab
Li et al. 2018b)	Mobility—tDCS	0%; 0.71	No	atDCS (affected M1), ctDCS (unaffected M1)	I: 1.5–2 mA; D: 7–20 min; ES: 35 cm^2^	10 weeks	TUG; 6 MWT; 10 MWT	Sham tDCS + rehabilitation
Li et al. 2018b)	Motor function—tDCS	82%; < 0.01	No	atDCS (affected M1), ctDCS (unaffected M1)	I: 2 mA; D: 10–25 min; ES: 7.07–35 cm^2^	6–10 weeks	Lower limb motricity index; MRC	Sham tDCS + physical therapy or rehabilitation
McIntyre et al. (2018)	Motor function—TMS	42%; NR	No	M1 (unaffected)	F: 1 Hz; T: 1; P: 240–1,500; MT (%): 90	10 weeks	MAS	Sham rTMS + physical therapy or occupational therapy
O'Brien et al. (2018)	Upper limb activity—TMS	67%; < 0.01	Not reported	M1 or PMd (unaffected or affected side)	F: 1–20 Hz; T: NR; P: 600–2,000; MT (%): 90–110	1–10 weeks	BBT; JTT; NHPT; PPT	Sham rTMS or Sham rTMS + motor training or CIMT or Brunnstrom hand manipulation
O'Brien et al. (2018)	Upper limb activity—tDCS	34%; 0.11	Not reported	M1 or PMd (unaffected or affected side)	I: 1–1.5 mA; D: 10–40 min; ES: 25–35 cm^2^	1–10 weeks	ARAT	Sham tDCS or Sham tDCS + occupational therapy or robot assisted training
Ghayour-Najafabadi et al. (2019)	Motor function—TMS	77%; < 0.01	No	M1 (unaffected or affected side) of the leg area or Cerebellum	F: 1–10 Hz; T: NR; P: 900–2,000; MT (%): 90–130	5–140	FMA-LL	Without stimulation or Sham rTMS or Sham rTMS + physical therapy or mirror therapy or rehabilitation
Ghayour-Najafabadi et al. (2019)	Mobility—TMS	0%; 0.62	No	M1 (unaffected or affected side) of the leg area or Cerebellum	F: 1–10 Hz; T: NR; P: 900–2,000; MT (%): 90–130	5–140	BBS; TUG	Without stimulation or Sham rTMS or Sham rTMS + physical therapy or mirror therapy or rehabilitation
Liu et al. (2019)	General neurological function—TMS	0%; 0.94	Yes (headache and anxiety)	LDLPFC	F: 10 Hz; T: NR; P: NR; MT (%): 80–110	10–20	NIHSS	Fluoxetine or citalopram or sertraline/Deanxit or Sham stimulation
Liu et al. (2019)	ADL—TMS	89%; < 0.01	Yes (headache and anxiety)	LDLPFC	F: 10 Hz; T: NR; P: NR; MT (%): 60–90	10–60	BI	General treatment or general treatment + citalopram/ fluoxetine
Tung et al. (2019)	Motor function—TMS	0%; 0.56	Yes (dizziness and tingling)	M1 (unaffected or affected side) of the leg area or Cerebellum or LDLPFC	F: 1–20 Hz; T: NR; P: 600–1,500; MT (%): 90–130	10–15	FMA-LL; Brunnstrom recovery stage for lower limb; plantar flexion peak torque; lower limb motricity index	Sham rTMS or Sham rTMS + task-oriented training or treadmill training or ankle strengthening exercise or movement therapy or physical therapy
Tung et al. (2019)	Mobility—TMS	35%; 0.18	Yes (dizziness and tingling)	M1 (unaffected or affected side) of the leg area or Cerebellum or LDLPFC	F: 1–10 Hz; T: NR; P: 600–1,000; MT (%): 90–110	5–40	BBS; FAC; Walking speed; ABMS II	Sham rTMS or Sham rTMS + task-oriented training or treadmill training or ankle strengthening exercise or movement therapy or physical therapy
van Lieshout et al. (2019)	Motor function—TMS	66%; < 0.01	NR	M1 or PMd (unaffected or affected side)	F: 1–5 Hz; T: NR; P: 240–1,800; MT (%): 80–120	5–24	FMA-UL	Sham rTMS + conventional therapy or virtual reality or physical therapy or functional task practice
van Lieshout et al. (2019)	Upper limb activity—TMS	49%; < 0.01	NR	M1 or PMd (unaffected or affected side)	F: 1–5 Hz; T: NR; P: 240–1,800; MT (%): 80–120	5–24	ARAT; JTT; BBT; NHPT; PPT	Sham rTMS + conventional therapy or virtual reality or physical therapy or functional task practice
Vaz et al. (2019)	Mobility—TMS	0%; 0.72	NR	M1 (unaffected or affected side) of the leg area	F: 1–10 Hz; T: NR; P: 600–2,000; MT (%): 90–100	10–30	10 MWT; 3–D gait analysis; 6 MWT; FAC; Motricity Index	Sham rTMS or Sham rTMS + physical therapy or task oriented training
Vaz et al. (2019)	Mobility—tDCS	25%;0.16	NR	M1 (unaffected or affected side) of the leg area	I: 1–2.5 mA; D: 7–20 min; ES: NR	7–12	10 MWT; 3-D gait analysis; 6 MWT; FAC	Sham tDCS + gait training or physical therapy
Xiang et al. (2019)	Motor function—TMS	0%; 0.68	Yes (headaches, fatigue, drowsiness, neck pain, anxiety, cast irritation, and neurocardiogenic syncope)	M1 (unaffected or affected side)	F: 1–25 Hz; T: NR; P: 150–1,800; MT (%): 80–130	1–24	BRS; JTT; NHPT; PPT; WMFT; FMA-LL	Sham rTMS
Xiang et al. (2019)	ADL—TMS	0%; 0.78	Yes (headaches, fatigue, drowsiness, neck pain, anxiety, cast irritation, and neurocardiogenic syncope)	M1 (unaffected or affected side)	F: 1–25 Hz; T: NR; P: 150–1,800; MT (%): 80–130	1–24	BI; activity index	Sham rTMS
Allida et al. (2020)	ADL—TMS	99%; < 0.01	No	LDLPFC or M1 (unaffected side)	F: 1–10 Hz; T: NR; P: 1,960; MT (%): 80%	10–28	BI	Sham rTMS + usual care
Allida et al. (2020)	General neurological function—TMS	93%; < 0.01	No	LDLPFC or RDLPFC or M1 (unaffected side)	F: 1–10 Hz; T: 20–50; P: 800–2,500; MT (%): 80–90	20–28	NIHSS	Sham or usual care
Elsner et al. (2020)	Upper limb activity—tDCS	0%; 0.84	No	M1 (affected or unaffected side)	I: 0.5–2 mA; D: 7–40 min; ES: NR	10–30	ARAT	Sham tDCS + physical therapy or occupational therapy or mirror therapy or virtual reality
Elsner et al. (2020)	Mobility—tDCS	31%; 0.14	No	M1 (affected or unaffected side)	I: 0.5–2 mA; D: 7–40 min; ES: NR	10–30	FAC; Walking velocity; Walking capacity	Sham tDCS + physical therapy or occupational therapy or mirror therapy or virtual reality
Elsner et al. (2020)	Motor function—tDCS	42%; 0.01	No	M1 (affected or unaffected side)	I: 0.5–2 mA; D: 7–40 min; ES: NR	10–30	MAL; FMA-UL	Sham tDCS + physical therapy or occupational therapy or mirror therapy or virtual reality
Elsner et al. (2020)	ADL—tDCS	0%; 0.87	No	M1 (affected or unaffected side)	I: 0.5–2 mA; D: 7–40 min; ES: NR	10–30	Barthel index; FIM	Sham tDCS + physical therapy or occupational therapy or mirror therapy or virtual reality
Kang et al. (2020)	Mobility—TMS	37%; NR	NR	M1 (unaffected or affected side) of the leg area or Cerebellum	F: 1–10 Hz; T: NR; P: 900–1,000; MT (%): 90–130	5–20	BBS; Tinetti test; Trunk control	Sham rTMS + physical therapy or mirror therapy or rehabilitation
Kang et al. (2020)	Mobility—tDCS	59%; NR	NR	M1 (unaffected or affected side)	I: 1–2 mA; D: 10–20 min; ES: 7.07–35 cm^2^	1–16	FMA/BBS	Sham tDCS + robotic training or physical therapy or rehabilitation or occupational therapy
Comino-Suárez et al. (2021)	Upper limb activity—tDCS	0%; 0.80	Yes (headache, fatigue, and tingling)	atDCS (affected M1), ctDCS (unaffected M1)	I: 1–2 mA; D: 7–30 min; ES: 25–35 cm^2^	2–36	FMA	Sham tDCS + robot assisted training or Lokomat or upper limb robotic assisted training
Comino-Suárez et al. (2021)	ADL—tDCS	0%; 0.66	Yes (headache, fatigue, and tingling)	atDCS (affected M1), ctDCS (unaffected M1)	I: 1–2 mA; D: 7–30 min; ES: 25–35 cm^2^	2–36	FMA	Sham tDCS + robot assisted training or Lokomat or upper limb robotic assisted training
Tien et al. (2020)	Mobility—tDCS	0%; 0.57	No	Non-cephalic areas; premotor cortex; M1 (unaffected or affected side)	I: 1–2 mA; D: 7–20 min; ES: 10–35 cm^2^	1–20	RAG; TRT; BBS	Sham tDCS
Comino-Suárez et al. (2021)	Motor function—tDCS	0%; 0.61	Yes (headache, fatigue, and tingling)	atDCS (affected M1), ctDCS (unaffected M1)	I: 1–2 mA; D: 7–30 min; ES: 25–35 cm^2^	2–36	FMA	Sham tDCS + robot assisted training or Lokomat or upper limb robotic assisted training
Reis et al. (2021)	Upper limb activity—tDCS	0%; 0.45	NR	NR	I: NR; D: 20–30 min; ES: NR	1–36	ARAT; BBT; WMFT	Sham + robotic assisted training
Shao et al. (2021)	General neurological function—TMS	33%; 0.23	No	NR	NR	7–20	Modified Scandinavian Stroke scale; modified Brunnstrom classification; NIHSS	Routine treatment or fluoxetine or Sham rTMS or Sham rTMS + Deanxit
Sun et al. (2021)	Motor function—tDCS	NR; NR	NR	Bihemispheric tDCS (affected and unaffected M1), atDCS (affected M1), ctDCS (affected M1)	I: 1–2 mA; D: 13–40 min; ES: 35 cm^2^	6–20	FMA-UL	Sham tDCS + rehabilitation or virtual reality or physical therapy or occupational therapy
Van Hoornweder et al. (2021)	Motor function—tDCS	68%; 0.01	NR	atDCS (affected M1, PMd & SMA), ctDCS (unaffected M1), bihemispheric tDCS (affected M1+unaffected M1)	I: 1–2 mA; D: 9–30 min; ES: 16–35 cm^2^	5–30	FMA-UL	Sham tDCS + CIMT or robot assisted training or virtual reality or occupational therapy
Huang et al. (2022)	Motor function—tDCS	87%; < 0.01	No	atDCS (affected M1), ctDCS (unaffected M1/S1)	I: 0.5–2 mA; D: 13–30 min; ES: 16–35 cm^2^	10–40	MAS	Sham tDCS + physical therapy or virtual reality or CIMT or robot-assisted training or electroacupuncture or exercise training
Krogh et al. (2022)	Mobility—TMS	0%; 0.62	NR	M1 (unaffected or affected side)	F: 1–10 Hz; T: 15–50; P: 900–2,000; AMT (%): 90–130	5–20	TUG, PASS, 10 MWT, BBS, gait velocity during non-standard gait analysis	Sham rTMS or Sham rTMS + motor imagery or physical training or treadmill training
Krogh et al. (2022)	Motor function—TMS	9%; 0.24	NR	M1 (unaffected or affected side)	F: 1–10 Hz; T: 15–50; P: 900–2,000; AMT (%): 90–130	5–20	FMA-LL	Sham rTMS or Sham rTMS + motor imagery or physical training or treadmill training
Ahmed et al. (2023)	Motor function—TMS	37%; 0.14	NR	M1 (unaffected or affected side)	F: 1–10 Hz; T: NR; P: NR; MT (%): NR	10–24	FMA	Sham rTMS + physical therapy or occupational therapy or rehabilitation
Ahmed et al. (2023)	ADL—TMS	84%; 0.01	NR	M1 (unaffected or affected side)	F: 1–10 Hz; T: NR; P: NR; MT (%): NR	10–24	BI, FIM, MAL	Sham rTMS + physical therapy or occupational therapy or rehabilitation
Ahmed et al. (2023)	Motor function—tDCS	81%; < 0.01	NR	atDCS (affected M1), ctDCS (unaffected M1)	I: 1–20 mA; D: 10–30 min; ES: NR	9–36	FMA-UL	Sham tDCS + robot assisted training or virtual reality or occupational therapy or physical therapy or CIMT
Ahmed et al. (2023)	ADL—tDCS	73%; 0.02	NR	atDCS (affected M1), ctDCS (unaffected M1)	I: 1–20 mA; D: 10–30 min; ES: NR	9–36	BI, FIM, MAL	Sham tDCS + robot assisted training or virtual reality or occupational therapy or physical therapy or CIMT
Chen X. et al. (2023)	Motor function—TMS	81%; < 0.01	Yes (headache and dizziness)	M1 (unaffected or affected side) of the leg area	F: 1–10 Hz; T: NR; P: NR; MT (%): 80–100	10–20	FMA-LL	Rehabilitation therapy + medical treatment or physical therapy + medical treatment or Sham rTMS + rehabilitation therapy + medical treatment
Chen X. et al. (2023)	General neurological function—TMS	68%; < 0.01	Yes (headache and dizziness)	M1 (unaffected or affected side) of the leg area	F: 1–10 Hz; T: NR; P: NR; MT (%): 80–100	10–20	NIHSS	Rehabilitation therapy + medical treatment or physical therapy + medical treatment or Sham rTMS + rehabilitation therapy + medical treatment
Chen X. et al. (2023)	ADL—TMS	97%; < 0.01	Yes (headache and dizziness)	M1 (unaffected or affected side) of the leg area	F: 1–10 Hz; T: NR; P: NR; MT (%): 80–100	10–20	BI	Rehabilitation therapy + medical treatment or
								physical therapy + medical treatment or Sham rTMS + rehabilitation therapy + medical treatment
Chen Y. et al. (2023)	ADL—TMS	84%; < 0.01	Yes (headaches, dizziness, palpitation, anxiety, gastrointestinal symptoms)	DLPFC (left or affected side)	F: 3–10 Hz; T: NR; P: NR; MT (%): 80–120	20–48	MBI; BI	Sham rTMS + cognitive training or routine medication treatment or rehabilitation or hyperbaric oxygen therapy or acupuncture or occupational therapy
Gao et al. (2023)	ADL—TMS	0%; NR	Yes (headache and dizziness)	DLPFC (left side)	F: 1–10 Hz; T: NR; P: 900–2,000; MT (%): 80–100	10–20	MBI	NR
Hofmeijer et al. (2023)	Mobility—TMS	69%; 0.02	NR	M1 (affected or unaffected side)	F: 1–10 Hz; T: NR; P: 1,000–1,200; MT (%): 90	5–21	FAC; BBS	Sham rTMS + rehabilitation or physical therapy or virtual reality
Hofmeijer et al. (2023)	Motor function—TMS	83%; < 0.01	NR	M1 (affected or unaffected side)	F: 1–10 Hz; T: NR; P: 100–1,800; MT (%): 80–90	10–24	FMA-UL	Sham rTMS + rehabilitation or physical therapy or virtual reality
Hofmeijer et al. (2023)	ADL—TMS	80% < 0.01	NR	M1 (affected or unaffected side)	F: 1–20 Hz; T: NR; P: 900–1,200; MT (%): 80–130	5–24	BI; FIM	Sham rTMS + rehabilitation or physical therapy or virtual reality
Lima et al. (2023)	Motor function—tDCS	76%; < 0.01	NR	M1 (affected or unaffected side)	I: NR; D: NR; ES: NR	NR	FMA-LL	Sham tDCS + PT or OT
Lima et al. (2023)	Mobility—tDCS	0%; 0.79	NR	M1 (affected or unaffected side)	I: NR; D: NR; ES: NR	NR	TUG; BBS	Sham tDCS + PT or robot assisted training
Xi et al. (2023)	Upper limb activity—TMS	0%; NR	NR	M1 (affected or unaffected side)	F: 1–20 Hz; T: NR; P: 1,200–1,500; MT (%): 90–110	8–20	BBT	Sham rTMS + task-oriented training
Xi et al. (2023)	Motor function—TMS	0%; NR	NR	M1 (affected or unaffected side); left posterior parietal cortex	F: 1–10 Hz; T: NR; P: 200–2,000; MT (%): 80–90	24–48	FMA-UL	Sham rTMS + task-oriented training or physical therapy or rehabilitation
Xi et al. (2023)	ADL—TMS	39.9%; NR	NR	M1 (affected or unaffected side); left posterior parietal cortex	F: 1–10 Hz; T: NR; P: 600–2,000; MT (%): 80–90	24–42	BI	Sham rTMS + task-oriented training
Xie et al. (2023)	ADL—TMS	36%; 0.21	No	DLPFC (Left, bilateral or unaffected side)	F: 1–10 Hz; T: NR; P: NR; MT (%): 80–120	20	MBI	Sham rTMS or no intervention
Zhou et al. (2023)	Mobility—TMS	0%; 0.99	No	M1 (affected and unaffected side); supplementary motor area; DLPFC; cerebellum	F: 0.5–50 Hz; T: NR; P: 450–3,000; MT (%): 80–130	1–15	BBS; TUG; Walking performance	Sham rTMS
Chen et al. (2024)	Motor function—TMS	79%; < 0.01	No	M1 (affected and unaffected side); cerebellum	F: 5 Hz; T: 20–40; P: 600–1,200; MT (%): 70–100	10–30	FMA-LL	Sham rTMS + rehabilitation or suspension exercise
	Mobility—TMS	78%; 0.01	No	M1 (affected and unaffected side); cerebellum	F: 5 Hz; T: 20–40; P: 600–1,200; MT (%): 70–100	10–30	BBS; TUG; 10 MWT	Sham rTMS + rehabilitation or suspension exercise
	ADL—TMS	0%; 0.80	No	M1 (affected and unaffected side); cerebellum	F: 5 Hz; T: 20–40; P: 600–1,200; MT (%): 80–100	10–20	MBI	Sham rTMS + rehabilitation or suspension exercise
Daoud et al. (2024)	ADL—TMS	0%; 0.48	No	DLPFC (left)	F: 5 Hz; T: 20–40; P: 600–1,200; MT (%): 56–80	20–30	BI; MBI	Sham rTMS or Sham rTMS + cognitive training
Chen et al. (2024)	Motor function—TMS	79%; < 0.01	No	M1 (affected and unaffected side); cerebellum	F: 5 Hz; T: 20–40; P: 600–1,200; MT (%): 70–100	10–30	FMA-LL	Sham rTMS + rehabilitation or suspension exercise
Jiang et al. (2024)	Motor function—TMS	65%; < 0.01	No	M1 (affected and/or unaffected side); cerebellum (ipsilesional)	F: 5 Hz; T: 1–40; P: 600–1,200; MT (%): 60–110	9–30	FMA-UL; FMA-LL; MAS	Sham TBS + PT or rehabilitation or virtual reality or RAT
	Upper limb activity—TMS	94%; < 0.01	No	M1 (affected or unaffected side)	F: 5 Hz; T: 1–20; P: 600; MT (%): 80–90	10	NHPT; ARAT	Sham TBS + PT or rehabilitation
	Mobility—TMS	59%; 0.08	No	M1 (affected and unaffected side); cerebellum (ipsilesional)	F: 5 Hz; T: 20–40; P: 600–1,200; MT (%): 80–100	10–30	BBS	Sham TBS + PT or rehabilitation
Lima et al. (2024)	Motor function—tDCS	0%; 1.00	No	M1 (affected and unaffected side)	I: 1–2 mA; D: 10–30 min; ES: NR	NR	FMA-UL	Sham tDCS + robot assisted training
Ren et al. (2024)	Motor function—TMS	86%; < 0.01	No	M1 (affected and unaffected side)	F: 1–10 Hz; T: NR; P: 900–1,000; MT (%): 80–120	15–30	FMA-UL	Sham rTMS
	Motor function—tDCS	0%; 0.75	No	M1 (affected and unaffected side)	I: NR; D: 20–30 min; ES: NR	5–20	FMA-UL	Sham tDCS
Tang et al. (2024)	Motor function—tDCS	49%; < 0.01	No	M1 (affected and unaffected side); supplementary motor area; DLPFC; cerebellum	I: 1–2 mA; D: 9–40 min; ES: 16–35 cm^2^	5–60	FMA-UL; ARAT	NR
	ADL—tDCS	37%; 0.07	No	M1 (affected and unaffected side)	I: 1.5–2 mA; D: 10–30 min; ES: 22–35 cm^2^	10–60	BI	NR
Wang J. et al. (2024)	Mobility—TMS	69%; < 0.01	Yes (Vertigo)	Cerebellum (contra or ipsilesional)	F: 1–10 Hz; T: NR; P: 600–1,200; MT (%): 80–100	10–21	BBS, TUG	Sham + PT or rehabilitation or mirror therapy
	ADL—TMS	80%; < 0.01	No	Cerebellum (contra or ipsilesional)	F: 5–10 Hz; T: 20–40; P: 600–1,200; MT (%): 80–110	10–24	BI, MBI	Rehabilitation or Sham rTMS + rehabilitation or Sham + PT or Sham + rehabilitation + acupuncture
Wang Y. et al. (2024)	Motor function—TMS	0%; NR	NR	M1 (affected and unaffected side)	F: 1–10 Hz; T: 1–NR; P: 1,000–1,200; MT (%): 80–90	15–40	FMA-UL	Rehabilitation or Sham rTMS + rehabilitation
	ADL—TMS	0%; NR	NR	M1 (affected and unaffected side)	F: 1–10 Hz; T: 1-NR; P: 1,000–1,200; MT (%): 80–90	15–40	BI, MBI	Rehabilitation or Sham rTMS + rehabilitation
Zeng et al. (2024)	Motor function—TMS	0%; 0.42	NR	Cerebellum (contralesional)	F: 1–10 Hz; T: 1-NR; P: 600–1,600; MT (%): 80–110	10–20	FMA-LL	Sham + PT or rehabilitation
	Mobility—TMS	76%; < 0.01	NR	Cerebellum (contra or ipsilesional)	F: 1–10 Hz; T: 1-NR; P: 600–1,000; MT (%): 80–110	5–21	BBS	Sham + PT or rehabilitation
Zhang J. J. et al. (2024)	ADL—tDCS	0%; 0.356	NR	M1 (unaffected side)	I: 2 mA; D: 20–30 min; ES: NR	10–15	MBI	Sham tDCS + VR or rehabilitation
Zhang N. et al. (2024)	Motor function -TMS	76.2%; < 0.01	NR	M1 (affected or unaffected side)	F: 5 Hz; T: 20–40; P: 600–1,200; MT (%): 60–110	9–30	FMA-UL	Sham + rehabilitation or PT or RAT or VR
	Upper limb activity—TMS	34.2%; 0.07	NR	M1 (affected or unaffected side)	F: 5 Hz; T: 20–40; P: 600–1,200; MT (%): 60–110	9–30	ARAT; WMFT; JTT	Sham + rehabilitation or PT or RAT or VR
Zhu et al. (2024)	ADL—TMS	52.6%; 0.12	No	DLPFC (left or unaffected side)	F: 1–10 Hz; T: 1–20; P: 600–1,000; MT (%): NR	5–30	MBI	NR
Barreto et al. (2025)	Motor function—TMS	81%; < 0.01	NR	M1 (affected or unaffected side); left posterior parietal cortex	F: 1–20 Hz; T: NR; P: 200–2,000; MT (%): 60–120	5–24	FMA-UL; WMFT; ARAT	Sham rTMS + physical therapy or occupational therapy or virtual reality or electrotherapy or Brunnstrom hand manipulation or CIMT or task oriented training
Jia et al. (2025)	Motor function—TMS	35%; 0.07	NR	M1 (affected and/or unaffected side); left DLPFC	F: 1–20 Hz; T: NR; P: 600–1,600; MT (%): 80–130	5–21	FMA-LL	NR
	Mobility—TMS	20%; 0.28	NR	M1 (affected and/or unaffected side); left DLPFC	F: 1–20 Hz; T: NR; P: 600–1,600; MT (%): 80–100	5–11	10 MWT; BBS	NR
Ma et al. (2025)	Motor function—TMS	87%; < 0.01	NR	M1 (affected and/or unaffected side); affected DLPFC	F: 1–10 Hz; T: NR; P: NR; MT (%): 80–100	10–20	NR	Sham rTMS or rehabilitation or acupuncture or ganglion block or cold water bath therapy
Usman et al. (2024)	Mobility—tDCS	0%; 0.89	No	M1 (affected and/or unaffected side)	I: 1–2 mA; D: 15–20 min; ES: 1.75–25 cm^2^	4–12	Gait speed	Sham tDCS or Sham tDCS + HIIT
Wang et al. (2025)	Mobility—TMS	20%; 0.25	No	Cerebellum (contra or ipsilesional)	F: 1–5 Hz; T: 1–40; P: 600–1,200; MT (%): 80–100	5–15	BBS; TUG; 10 MWT	Sham + PT
	Motor function—TMS	67%; 0.05	No	Cerebellum (contralesional)	F: 5 Hz; T: 20–40; P: 600–1,200; MT (%): 80	10	FMA-LL	Sham + PT
	ADL—TMS	0%; 0.67	No	Cerebellum (contralesional)	F: 5 Hz; T: 20–40; P: 600–1,200; MT (%): 80	10–20	BI, MBI	Sham + PT or rehabilitation
Xie et al. (2025)	Motor function—TMS	35%; 0.02	Yes (seizure, headache, drowsiness)	M1 (affected and/or unaffected side); left DLPFC; premotor cortex (contralateral); cerebellum (ipsilateral)	F: 0.1–20 Hz; T: NR; P: 200–7,500; MT (%): 80–130	5–40	FMA-UL; FMA-LL	Sham + rehabilitation or PT or OT or rehabilitation
	General neurological function—TMS	81%; < 0.01	Yes (seizure, headache, drowsiness)	M1 (affected and/or unaffected side)	F: 1–10 Hz; T: NR; P: 900–1,800; MT (%): 80–120	5–20	NIHSS	Sham + rehabilitation or PT
	ADL—TMS	83%; < 0.01	Yes (seizure, headache, drowsiness)	M1 (affected and/or unaffected side)	F: 0.5–10 Hz; T: NR; P: 200–1,200; MT (%): 80–130	5–40	MBI	Sham + rehabilitation or PT or OT or rehabilitation
Yu et al. (2025)	Motor function—tDCS	86%; < 0.01	Yes (headache, tingling, burning, itching)	M1 (affected side)	I: 1–2 mA; D: 20–45 min; ES: 25–50 cm^2^	5–36	FMA-UL	Sham tDCS + rehabilitation or acupuncture or PT or RT
	Upper limb activity—tDCS	17%; 0.30	No	M1 (affected side)	I: 1–2 mA; D: 20–45 min; ES: 25–50 cm^2^	12–24	WMFT	Sham tDCS + rehabilitation or acupuncture or PT
	ADL—tDCS	88%; < 0.01	Yes (headache, tingling, burning, itching)	M1 (affected side)	I: 1–2 mA; D: 20–45 min; ES: 25–50 cm^2^	5–36	BI	Sham tDCS + rehabilitation or acupuncture or PT or RT
Zhang et al. (2025)	Motor function—TMS	86%; < 0.01	NR	M1 (affected and unaffected side); cerebellum (ipsilesional); premotor cortex (unaffected side); S1 (affected side)	F: 1–20 Hz; T: NR; P: 500–2,000; MT (%): 20–120	5–20	FMA-UL	Sham + PT or OT

10 MWT, 10-meter walking test; 6 MWT, 6-minute walk test; 9 HPT, 9-Hole Peg test; AT, the albert test; ABMS-II, ability for basic movement scale II; ARAT, action research arm test; AS, Ashworth spasticity; b-tDCS, bilateral-tDCS; BDLPFC, bilateral dorsolateral prefrontal cortex; Bi, bilateral (anodal + cathodal); B&B, box and block test; BBS, Berg balance scale; BBT, box and block test; BI, Barthel index; BRS, Brunnstrom recovery stages; C3/C4/F3, according to the 10–20 international electroencephalography system; CIMT, constraint-induced movement therapy; cTBS, continuous theta burst stimulation; D, duration of stimulation; ES, electrodes size; F, frequency; FAC, functional ambulation category; FIM, functional independence measure; FMA, Fugl-Meyer assessment scale; FMA-LL, Fugl-Meyer assessment scale lower limb; FMA-UL, Fugl-Meyer assessment scale upper limb; FTP, functional task practice; FTT, finger tapping test; HF-rTMS, high frequency repetitive transcranial magnetic stimulation; I, current intensity; iTBS, intermittent theta burst stimulation; JTT, Jebsen Taylor test; l-DLPFC, left dorsolateral prefrontal cortex; LF-rTMS, high frequency repetitive transcranial magnetic stimulation; LLMI, lower limb motricity index; MEPs, motor evoked potentials; HADS, hospital anxiety and depression scale; MAL, motor activity log; M1, primary motor cortex; MAS, modified Ashworth scale; MRC, medical research council motor power score; mSSS, modified Scandinavian stroke scale; MT, motor training; MT (%), percentage of motor threshold; MFT, manual function test; NHPT, nine Hole Peg test; NIHSS, national institutes of health stroke scale; NMES, neuromuscular electrical stimulation; NA, not applied; NNT, number needed to treat; NR, not reported; NEADL, Nottingham extended activities of daily living scale; OT, occupational therapy; P, pulses per train; PASS, postural assessment scale for stroke patients; PMd, dorsal premotor cortex; PPT, Purdue pegboard test; PT, physical therapy; PS, pinch strength; RAAT, robot assisted arm training; RAGT, robot-assisted gait training; RAT, robot assisted training; r-DLPFC, right dorsolateral prefrontal cortex; RT, rehabilitation treatment; RNS, vagus nerve stimulation; ROM, range of motion; RS, Rankin scale; S1, primary sensory cortex; SIS, stroke impact scale; SIAS, stroke impairment assessment set; T, number of trains; TRT, task-related training; TUG, timed up-and-go test; VR, virtual reality; WMFT, wolf motor function test; TI, Tinetti test; TIS, trunk impairment scale; LDLPFC, left dorsolateral prefrontal cortex; RDLPFC, right dorsolateral prefrontal cortex.

### 3.2 Results of the methodological quality (AMSTAR)

AMSTAR scores ranged from 8 (McIntyre et al., 2018; Kang et al., 2020; Sun et al., 2021) to 16 points (Allida et al., 2020; Elsner et al., 2020; Barreto et al., 2025). Twenty three studies (41.1%) were considered to be of high quality (Elsner et al., 2016, 2020; O'Brien et al., 2018; Liu et al., 2019; Vaz et al., 2019; Allida et al., 2020; Comino-Suárez et al., 2021; Reis et al., 2021; Shao et al., 2021; Huang et al., 2022; Krogh et al., 2022; Gao et al., 2023; Lima et al., 2023; Xie et al., 2023; Jiang et al., 2024; Lima et al., 2024; Zhang N. et al., 2024; Tang et al., 2024; Usman et al., 2024; Zeng et al., 2024; Barreto et al., 2025; Jia et al., 2025), 25 studies (44.6%) were considered to be of moderate quality (Graef et al., 2016; Tedesco Triccas et al., 2016; Zhang et al., 2017a; Li et al., 2018a,b; Ghayour-Najafabadi et al., 2019; Tung et al., 2019; van Lieshout et al., 2019; Xiang et al., 2019; Tien et al., 2020; Hofmeijer et al., 2023; Xi et al., 2023; Chen X. et al., 2023; Zhou et al., 2023; Chen et al., 2024; Daoud et al., 2024; Zhang J. J. et al., 2024; Wang J. et al., 2024; Ren et al., 2024; Wang Y. et al., 2024; Wang et al., 2025; Ma et al., 2025; Xie et al., 2025; Zhang et al., 2025), two study (3.6%) were considered as “low quality”(Zhang et al., 2017b; Zhu et al., 2024), and 6 studies (10.7%) were classified as critically low quality (McIntyre et al., 2018; Kang et al., 2020; Sun et al., 2021; Ahmed et al., 2023; Chen Y. et al., 2023; Yu et al., 2025).

The items with the highest scores across reviews were: “PICO Components” (item 1); “study designs for inclusion in the review” (item 3); “perform data extraction in duplicate” (item 6); risk of bias (RoB) in individual studies that were included in the review” (item 9); “appropriate methods for statistical combination of results” (item 11); “quantitative synthesis did the review authors carry out an adequate investigation of publication bias” (item 15) and “potential sources of conflict of interest, including any funding they received for conducting the review” (item 16). Conversely, the items with the highest proportion of studies presenting risk of bias were “whether the review and report justified any significant deviation from the protocol” (item 2); “authors use a comprehensive literature search strategy” (item 4) and “funding for the studies included in the review” (item 10). The AMSTAR ratings are presented in Table 2.

**Table 2:** AMSTAR ratings.

**Study**	**1**	2^*^	**3**	4^*^	**5**	**6**	7^*^	**8**	9^*^	**10**	11^*^	**12**	**13**	**14**	15^*^	**16**	**Total score (classification of quality)**
Elsner et al. (2016)	Y	Y	Y	PY	Y	Y	Y	Y	Y	N	Y	Y	Y	Y	Y	Y	14 points (high)
Graef et al. (2016)	Y	N	Y	PY	Y	Y	Y	Y	Y	N	Y	Y	Y	N	Y	Y	12 points (low)
Tedesco Triccas et al. (2016)	Y	N	Y	PY	N	Y	Y	Y	Y	Y	Y	Y	Y	Y	Y	Y	13 points (moderate)
Shen et al. (2017)	Y	Y	Y	PY	Y	Y	Y	Y	Y	N	Y	Y	Y	Y	Y	Y	14 points (high)
Zhang et al. 2017a)	Y	Y	Y	PY	Y	Y	Y	PY	Y	N	Y	Y	N	Y	Y	Y	12 points (moderate)
Zhang et al. 2017b)	Y	N	Y	PY	Y	Y	N	Y	Y	N	Y	Y	Y	Y	Y	Y	12 points (low)
Li et al. 2018a)	Y	N	Y	PY	Y	Y	Y	Y	Y	N	Y	Y	Y	N	Y	Y	13 points (moderate)
Li et al. 2018b)	Y	N	Y	PY	Y	Y	Y	Y	Y	N	Y	Y	Y	Y	Y	N	12 points (moderate)
McIntyre et al. (2018)	Y	N	Y	PY	N	Y	N	Y	Y	N	N	N	Y	N	Y	Y	8 points (critically low)
O'Brien et al. (2018)	Y	Y	Y	PY	Y	Y	Y	Y	Y	N	Y	Y	Y	N	Y	Y	13 points (high)
Ghayour-Najafabadi et al. (2019)	Y	Y	Y	PY	Y	Y	Y	Y	Y	Y	N	Y	Y	Y	Y	Y	14 points (moderate)
Liu et al. (2019)	Y	Y	Y	PY	Y	Y	Y	Y	Y	N	Y	Y	Y	Y	Y	Y	14 points (high)
Tung et al. (2019)	Y	N	Y	PY	Y	Y	Y	Y	Y	N	Y	Y	Y	Y	Y	Y	13 points (moderate)
van Lieshout et al. (2019)	Y	N	Y	PY	Y	Y	Y	Y	Y	N	Y	Y	Y	Y	Y	Y	13 points (moderate)
Vaz et al. (2019)	Y	Y	Y	PY	Y	Y	Y	Y	Y	Y	Y	Y	Y	Y	Y	Y	15 points (high)
Xiang et al. (2019)	Y	N	Y	PY	Y	Y	Y	PY	Y	N	Y	Y	Y	Y	Y	Y	12 points (moderate)
Allida et al. (2020)	Y	Y	Y	Y	Y	Y	Y	Y	Y	Y	Y	Y	Y	Y	Y	Y	16 points (high)
Elsner et al. (2020)	Y	Y	Y	Y	Y	Y	Y	Y	Y	Y	Y	Y	Y	Y	Y	Y	16 points (high)
Kang et al. (2020)	Y	N	Y	PY	N	Y	Y	PY	N	N	Y	N	N	Y	Y	Y	8 points (critically low)
Tien et al. (2020)	Y	N	Y	PY	Y	Y	Y	Y	Y	N	Y	N	Y	Y	Y	Y	12 points (moderate)
Comino-Suárez et al. (2021)	Y	Y	Y	PY	Y	Y	Y	Y	Y	N	Y	Y	Y	N	Y	Y	13 points (high)
Reis et al. (2021)	Y	Y	Y	Y	Y	Y	Y	Y	Y	N	Y	Y	Y	Y	Y	Y	15 points (high)
Shao et al. (2021)	Y	Y	Y	PY	Y	Y	Y	Y	Y	N	Y	Y	Y	Y	Y	Y	14 points (high)
Sun et al. (2021)	Y	N	Y	PY	Y	N	Y	PY	Y	N	Y	N	N	N	Y	Y	8 points (critically low)
Van Hoornweder et al. (2021)	Y	N	Y	PY	Y	Y	Y	Y	Y	N	Y	Y	Y	Y	Y	Y	13 points (moderate)
Huang et al. (2022)	Y	Y	Y	PY	Y	Y	Y	Y	Y	N	Y	Y	Y	Y	Y	Y	14 points (high)
Krogh et al. (2022)	Y	PY	Y	Y	Y	Y	Y	Y	Y	N	Y	Y	Y	Y	Y	Y	14 points (high)
Ahmed et al. (2023)	Y	N	Y	PY	Y	Y	Y	PY	Y	N	Y	N	N	Y	Y	Y	10 points (critically low)
Chen X. et al. (2023)	Y	Y	Y	PY	Y	Y	Y	Y	Y	N	Y	Y	N	N	Y	Y	12 points (moderate)
Chen Y. et al. (2023)	Y	N	Y	PY	Y	Y	Y	PY	Y	N	Y	Y	Y	N	N	Y	10 points (critically low)
Gao et al. (2023)	Y	Y	Y	PY	Y	Y	Y	PY	Y	N	Y	Y	Y	Y	Y	Y	13 points (high)
Hofmeijer et al. (2023)	Y	Y	Y	PY	Y	N	Y	Y	Y	N	Y	Y	Y	Y	Y	Y	13 points (moderate)
Lima et al. (2023)	Y	Y	Y	Y	Y	Y	Y	Y	Y	N	Y	Y	Y	Y	Y	Y	15 points (high)
Xi et al. (2023)	Y	Y	Y	PY	N	Y	N	Y	Y	N	Y	Y	Y	Y	Y	Y	12 points (moderate)
Xie et al. (2023)	Y	Y	Y	PY	Y	Y	Y	PY	Y	N	Y	Y	Y	Y	Y	Y	13 points (high)
Zhou et al. (2023)	Y	N	Y	PY	N	Y	Y	Y	Y	N	Y	Y	Y	Y	Y	Y	12 points (moderate)
Chen et al. (2024)	Y	Y	Y	PY	Y	Y	Y	Y	Y	Y	Y	N	N	Y	Y	Y	13 points (moderate)
Daoud et al. (2024)	Y	Y	Y	PY	N	N	Y	Y	Y	N	Y	Y	N	Y	Y	Y	11 points (moderate)
Jiang et al. (2024)	Y	Y	Y	PY	Y	Y	Y	PY	Y	N	Y	Y	Y	Y	Y	Y	13 points (high)
Lima et al. (2024)	Y	Y	Y	Y	Y	Y	Y	PY	Y	N	Y	Y	Y	Y	Y	Y	14 points (high)
Ren et al. (2024)	Y	Y	Y	PY	Y	Y	Y	Y	Y	N	Y	N	N	Y	Y	Y	12 points (moderate)
Tang et al. (2024)	Y	Y	Y	PY	N	Y	Y	PY	Y	N	Y	Y	Y	Y	Y	Y	12 points (high)
Wang J. et al. (2024)	Y	N	Y	PY	Y	Y	Y	PY	Y	N	Y	Y	Y	Y	Y	Y	12 points (moderate)
Wang Y. et al. (2024)	Y	Y	Y	PY	Y	Y	Y	Y	Y	Y	N	Y	Y	Y	Y	Y	14 points (moderate)
Zeng et al. (2024)	Y	Y	Y	PY	Y	Y	Y	Y	Y	N	Y	Y	Y	Y	Y	Y	14 points (high)
Zhang J. J. et al. (2024)	Y	N	Y	PY	Y	Y	Y	PY	Y	N	Y	Y	Y	Y	Y	Y	13 points (high)
Zhang N. et al. (2024)	Y	Y	Y	PY	Y	Y	Y	Y	Y	N	Y	Y	Y	N	N	Y	12 points (moderate)
Zhu et al. (2024)	Y	Y	Y	PY	N	Y	Y	PY	Y	N	Y	N	N	N	Y	Y	9 points (low)
Barreto et al. (2025)	Y	Y	Y	Y	Y	Y	Y	Y	Y	Y	Y	Y	Y	Y	Y	Y	16 points (high)
Jia et al. (2025)	Y	Y	Y	PY	Y	Y	Y	PY	Y	N	Y	Y	Y	Y	Y	Y	13 points (high)
Ma et al. (2025)	Y	Y	Y	PY	Y	Y	Y	PY	Y	N	Y	N	N	Y	Y	Y	11 points (moderate)
Usman et al. (2024)	Y	Y	Y	Y	Y	Y	Y	PY	Y	N	Y	Y	Y	Y	Y	Y	14 points (high)
Wang et al. (2025)	Y	Y	Y	PY	Y	Y	Y	Y	Y	N	Y	Y	N	Y	Y	Y	13 points (moderate)
Xie et al. (2025)	Y	Y	Y	PY	Y	Y	Y	Y	Y	N	N	Y	Y	Y	Y	Y	13 points (moderate)
Yu et al. (2025)	Y	N	Y	PY	Y	Y	Y	PY	Y	N	Y	Y	N	N	Y	Y	10 points (critically low)
Zhang et al. (2025)	Y	Y	Y	PY	Y	N	Y	PY	Y	N	Y	Y	Y	Y	N	Y	12 points (moderate)

^*^Critically points; Y, yes; N, no; PY, partially yes.

### 3.3 Grading of evidence results (GRADE)

Based on the GRADE assessment, we categorized the evidence according to each NIBS technique. Tables 3a, 3b present a Summary of Findings (SoF) and evidence quality for each meta-analysis on rTMS for body structure/function and activity domains, respectively, while Tables 4a, 4b provide the same for tDCS meta-analyses.

**Table 3a:** Summary of findings (SoF) and certainty of evidence regarding included studies that investigated the effects of repetitive transcranial magnetic stimulation (rTMS) in ICF body structure and function domains.

**Certainty assessment**							**No. of patients**		**Effect**		**Certainty**

**No. of studies**	**Study design**	**Risk of bias**	**Inconsistency**	**Indirectness**	**Imprecision**	**Other considerations**	**rTMS**	**Sham rTMS**	**Relative (95% CI)**	**Absolute (95% CI)**	
**Shen et al. (** **2017** **)—General neurological function**											
7	Randomized trials	Not serious	Serious^a^	Serious^b^	Serious^c^	None	164	165	–	SMD **0.94 SD lower** (1.29 lower to 0.6 lower)	⊕○○○ Very low^a, b, c^
**Liu et al. (** **2019** **)—General neurological function**											
4	Randomized trials	Not serious	Not serious	Serious^d^	Serious^e^	None	111	110	–	SMD **0.91 SD lower** (1.19 lower to 0.63 lower)	⊕⊕○○ Low^d, e^
**Allida et al. (** **2020** **)—General neurological function**											
3	Randomized trials	Not serious	Serious^f, g^	Serious^b^	Serious^h^	None	145	145	–	SMD **2.21 SD lower** (3.32 lower to 1.09 lower)	⊕○○○ Very low^b, f, g, h^
**Shao et al. (** **2021** **)—General neurological function**											
3	Randomized trials	Not serious	Not serious	Not serious	Very serious^h, i^	None	68	68	–	SMD **0.67 SD lower** (1.02 lower to 0.32 lower)	⊕⊕○○ Low^h, i^
**Chen X. et al. (** **2023** **)—General neurological function**											
12	Randomized trials	Serious^j^	Very serious^a, f^	Serious^b^	Not serious	None	278	180	–	SMD **0.94 SD lower** (1.33 lower to 0.54 lower)	⊕○○○ Very low^a, b, f, j^
**Xie et al. (** **2025** **)—General neurological function**											
12	Randomized trials	Serious^j^	Very serious^a, f^	Not serious	Very serious^i, k^	None	351	299	–	SMD **0.33 SD lower** (0.71 lower to 0.05 higher)	⊕○○○ Very low^a, f, i, j, k^
**Graef et al. (** **2016** **)—Motor function**											
4	Randomized trials	Serious^j^	Serious^f^	Not serious	Very serious^k, l^	None	83	61	–	SMD **0.04 SD lower** (0.38 lower to 0.3 higher)	⊕○○○ Very low^f, j, k, l^
8	Randomized trials	Serious^j^	Serious^f^	Not serious	Serious^l^	None	150	151	–	SMD **0.29 SD lower** (0.64 lower to 0.06 higher)	⊕○○○ Very low^f, j, l^
**Zhang et al. (** **2017b** **)—Motor function**											
27	Randomized trials	Very serious^m^	Very serious^a, f^	Not serious	Serious^n^	None	470	199	–	SMD **0.49 SD lower** (0.68 lower to 0.29 lower)	⊕○○○ Very low^a, f, m, n^
**Li et al. (** **2018a** **)—Motor function**											
3	Randomized trials	Serious^j^	Not serious	Not serious	Very serious^h, k^	None	38	38	–	SMD **0.43 SD lower** (0.56 lower to 0.3 higher)	⊕○○○ Very low^h, j, k^
**McIntyre et al. (** **2018** **)—Motor function**											
2	Randomized trials	Very serious°	Very serious^p, q^	Not serious	Very serious^k, l^	None	28	28	–	SMD **0.34 SD lower** (0.97 lower to 0.3 higher)	⊕○○○ Very low^k, l, o, p, q^
**Ghayour-Najafabadi et al. (** **2019** **)—Motor function**											
6	Randomized trials	Serious^j^	Very serious^f, p^	Not serious	Very serious^h, k^	None	93	84	–	SMD **0.01 SD lower** (0.31 lower to 0.29 higher)	⊕○○○ Very low^f, h, j, k, p^
**Tung et al. (** **2019** **)—Motor function**											
7	Randomized trials	Serious^j^	Serious^f, p^	Not serious	Serious^h, i^	None	73	70	–	SMD **0.66 SD lower** (1 lower to 0.32 lower)	⊕○○○ Very low^f, h, i, j, p^
15	Randomized trials	Serious^j^	Very serious^f, p^	Not serious	Very serious^h, i^	None	289	244	–	SMD **0.46 SD lower** (0.84 lower to 0.09 lower)	⊕○○○ Very low^f, h, i, j, p^
**Xiang et al. (** **2019** **)—Motor function**											
43	Randomized trials	Serious^j^	Serious^p, q^	Not serious	Not serious	None	739	743	–	SMD **0.5 SD lower** (0.6 lower to 0.39 lower)	⊕⊕○○ Low^j, p, q^
**Krogh et al. (** **2022** **)—Motor function**											
5	Randomized trials	Not serious	Serious^a, f^	Not serious	Very serious^k, l^	None	77	78	–	SMD **0.19 SD lower** (0.51 lower to 0.13 higher)	⊕○○○ Very low^a, f, k, l^
**Ahmed et al. (** **2023** **)—Motor function**											
8	Randomized trials	Very serious°	Very serious^a, f^	Not serious	Serious^k^	None	246	169	–	SMD **0.04 SD lower** (0.24 lower to 0.16 higher)	⊕○○○ Very low^a, f, k, o^
**Chen X. et al. (** **2023** **)—Motor function**											
8	Randomized trials	Serious^j^	Very serious^a, f^	Serious^b^	Not serious	None	330	217	–	SMD **1.22 SD lower** (1.7 lower to 0.73 lower)	⊕○○○ Very low^a, b, f, j^
**Hofmeijer et al. (** **2023** **)—Motor function**											
10	Randomized trials	Serious^j^	Very serious^a, f^	Not serious	Serious^n^	None	323	219	–	SMD **0.94 SD lower** (1.43 lower to 0.45 lower)	⊕○○○ Very low^a, f, j, n^
**Xi et al. (** **2023** **)—motor function**											
8	Randomized trials	Serious^j^	Serious^a, f^	Not serious	Not serious	None	245	241	–	SMD **1.07 SD lower** (0.88 lower to 1.25 lower)	⊕⊕○○ Low^a, f, j^
**Chen et al. (** **2024** **)—Motor function**											
5	Randomized trials	Serious^j^	Serious^f^	Not serious	Very serious^c, k^	None	83	83	–	SMD **0.37 SD lower** (1.07 lower to 0.34 higher)	⊕○○○ Very low^c, f, j, k^
18	Randomized trials	Not serious	Very serious^a, f^	Not serious	Not serious	None	226	219	–	SMD **0.59 SD lower** (0.93 lower to 0.25 lower)	⊕⊕○○ Low^a, f^
**Ren et al. (** **2024** **)—Motor function**											
2	Randomized trials	Serious^j^	Very serious^a, f^	Not serious	Very serious^c, k^	None	36	34	–	SMD **0.83 SD lower** (2.16 lower to 0.51 higher)	⊕○○○ Very low^a, c, f, j, k^
**Wang Y. et al. (** **2024** **)—Motor function**											
3	Randomized trials	Serious^j^	Serious^a^	Not serious	Serious^c^	None	50	44	–	SMD **0.89 SD lower** (1.31 lower to 0.48 lower)	⊕○○○ Very low^a, c, j^
**Zeng et al. (** **2024** **)—Motor function**											
4	Randomized trials	Not serious	Very serious^a, f^	Not serious	Serious^c^	None	93	94	–	SMD **0.89 SD lower** (1.19 lower to 0.58 lower)	⊕○○○ Very low^a, c, f^
**Zhang J. J. et al. (** **2024** **)—Motor function**											
14	Randomized trials	Serious^j^	Very serious^a, f^	Serious^r^	Serious^c^	None	197	182	–	SMD **0.65 SD lower** (1.08 lower to 0.21 lower)	⊕○○○ Very low^a, c, f, j, r^
**Barreto et al. (** **2025** **)—Motor function**											
35	Randomized trials	Not serious	Serious^a, f^	Not serious	Serious^n^	None	897	700	–	SMD **0.57 SD lower** (0.82 lower to 0.32 lower)	⊕⊕○○ Low^a, f, n^
**Jia et al. (** **2025** **)—Motor function**											
18	Randomized trials	Not serious	Very serious^a, f^	Not serious	Not serious	None	362	361	–	SMD **0.45 SD lower** (0.65 lower to 0.25 lower)	⊕⊕○○ Low^a, f^
**Ma et al. (** **2025** **)—Motor function**											
10	Randomized trials	Serious^j^	Very serious^a, s^	Not serious	Not serious	None	255	259	–	SMD **1.14 SD lower** (1.69 lower to 0.58 lower)	⊕○○○ Very low^a, j, s^
3	Randomized trials	Serious^j^	Serious^f^	Not serious	Very serious^c, k^	None	45	45	–	SMD **0.38 SD lower** (0.81 lower to 0.04 higher)	⊕○○○ Very low^c, f, j, k^
**Xie et al. (** **2025** **)—Motor function**											
36	Randomized trials	Serious^j^	Very serious^a, f^	Not serious	Not serious	None	878	776	–	SMD **0.49 SD lower** (0.59 lower to 0.39 lower)	⊕○○○ Very low^a, f, j^
**Zhang et al. (** **2025** **)—Motor function**											
37	Randomized trials	Serious^j^	Very serious^a, f^	Not serious	Not serious	None	773	712	–	SMD **0.65 SD lower** (0.95 lower to 0.36 lower)	⊕○○○ Very low^a, f, j^

CI, confidence interval; SMD, standardized mean difference.

^a^There is a high variability between the rTMS protocols in included studies.

^b^Control groups of the some included studies comprised medications intake.

^c^The sample size of the present meta-analysis was smaller than the required.

^d^Control groups of the some included studies comprised medications intake.

^e^The sample size of the present meta-analysis was smaller than the required.

^f^There is a critical difference in time since stroke of populations included in different studies.

^g^There is a high variability between the rTMS protocols in included studies.

^h^The sample size of the present meta-analysis was smaller than the required.

^i^The meta-analysis effect size did not cross the mCID cut-off.

^j^The meta-analysis study was classified as “moderate” according to the AMSTAR assessment.

^k^Imprecise due to the diamond touches the null line.

^l^The sample size of the present meta-analysis was smaller than the required.

^m^The meta-analysis study was classified as “low quality” according AMSTAR assessment.

^n^The meta-analysis presented a high variability in effect size among the included studies.

^o^The meta-analysis study was classified as “critically low quality” according AMSTAR assessment.

^p^There is a high variability between the rTMS protocols in included studies.

^q^There is a high variability between the design in included studies of the meta-analysis.

^r^Control groups of the some included studies comprised other neuromodulation approaches.

^s^There is a lack of information about stroke characteristics and outcomes of the included studies.

**Table 3b:** Summary of findings (SoF) and certainty of evidence regarding included studies that investigated the effects of repetitive transcranial magnetic stimulation (rTMS) in ICF activity and participation domains.

**Certainty assessment**							**No. of patients**		**Effect**		**Certainty**

**No. of studies**	**Study design**	**Risk of bias**	**Inconsistency**	**Indirectness**	**Imprecision**	**Other considerations**	**rTMS**	**Sham rTMS**	**Relative (95% CI)**	**Absolute (95% CI)**	
**Shen et al. (** **2017** **)—Activities of daily living**											
7	Randomized trials	Not serious	Serious^a^	Serious^b^	Not serious	None	331	329	–	SMD **1.2 SD lower** (1.72 lower to 0.68 lower)	⊕⊕○○ Low^a, b^
**Liu et al. (** **2019** **)—Activities of daily living**											
3	Randomized trials	Not serious	Not serious	Serious^b^	Serious^c^	None	157	156	–	SMD **1.09 SD lower** (1.84 lower to 0.34 lower)	⊕⊕○○ Low^b, c^
**Xiang et al. (** **2019** **)—Activities of daily living**											
7	Randomized trials	Serious^d^	Not serious	Not serious	Not serious	None	212	213	–	SMD **0.82 SD lower** (1.05 lower to 0.59 lower)	⊕⊕⊕○ Moderate^d^
**Allida et al. (** **2020** **)—Activities of daily living**											
2	Randomized trials	Not serious	Not serious	Serious^b^	Very serious^e, f^	None	104	104	–	SMD **1.84 SD higher** (5.08 higher to 1.4 lower)	⊕○○○ Very low^b, e, f^
**Ahmed et al. (** **2023** **)—Activities of daily living**											
2	Randomized trials	Very serious^g^	Very serious^a, h^	Not serious	Serious^c^	None	65	63	–	SMD **0.4 SD lower** (0.76 lower to 0.04 lower)	⊕○○○ Very low^a, c, g, h^
**Chen X. et al. (** **2023** **)—Activities of daily living**											
8	Randomized trials	Serious^d^	Serious^a^	Serious^b^	Serious^c^	None	205	125	–	SMD **1.28 SD lower** (1.55 lower to 1.02 lower)	⊕○○○ Very low^a, b, c, d^
**Chen Y. et al. (** **2023** **)—Activities of daily living**											
10	Randomized trials	Very serious^g^	Not serious	Not serious	Not serious	None	330	328	–	SMD **1.15 SD lower** (1.57 lower to 0.73 lower)	⊕⊕○○ Low^g^
**Gao et al. (** **2023** **)—Activities of daily living**											
4	Randomized trials	Not serious	Very serious^a, h^	Not serious	Serious^c^	None	61	56	–	SMD **0.42 SD lower** (0.06 lower to 0.78 lower)	⊕○○○ Very low^a, c, h^
**Hofmeijer et al. (** **2023** **)—Activities of daily living**											
8	Randomized trials	Serious^d^	Very serious^a, h^	Not serious	Serious^c^	None	160	105	–	SMD **1.72 SD lower** (2.48 lower to 0.96 lower)	⊕○○○ Very low^a, c, d, h^
**Xi et al. (** **2023** **)—Activities of daily living**											
3	Randomized trials	Serious^d^	Very serious^a, h^	Not serious	Serious^c^	None	100	97	–	SMD **0.74 SD lower** (1.03 lower to 0.45 lower)	⊕○○○ Very low^a, c, d, h^
**Xie et al. (** **2023** **)—Activities of daily living**											
3	Randomized trials	Not serious	Serious^a^	Not serious	Very serious^c, f^	None	58	56	–	SMD **0.03 SD lower** (0.5 lower to 0.44 higher)	⊕○○○ Very low^a, c, f^
**Chen et al. (** **2024** **)—Activities of daily living**											
3	Randomized trials	Serious^d^	Serious^h^	Not serious	Very serious^c, f^	None	47	47	–	SMD **0.31 SD lower** (0.72 lower to 0.1 higher)	⊕○○○ Very low^c, d, f, h^
**Daoud et al. (** **2024** **)—Activities of daily living**											
4	Randomized trials	Serious^d^	Serious^h^	Not serious	Serious^c^	None	120	117	–	SMD **0.82 SD lower** (1.08 lower to 0.55 lower)	⊕○○○ Very low^c, d, h^
**Wang J. et al. (** **2024** **)—Activities of daily living**											
6	Randomized trials	Serious^d^	Very serious^a, h^	Not serious	Serious^c^	None	140	140	–	SMD **0.83 SD lower** (1.41 lower to 0.25 lower)	⊕○○○ Very low^a, c, d, h^
**Wang Y. et al. (** **2024** **)—Activities of daily living**											
3	Randomized trials	Serious^d^	Serious^a^	Not serious	Serious^e^	None	50	44	–	SMD **0.92 SD lower** (1.34 lower to 0.51 lower)	⊕○○○ Very low^a, d, e^
**Wang et al. (** **2025** **)—Activities of daily living**											
4	Randomized trials	Serious^d^	Serious^h^	Not serious	Very serious^c, i^	None	60	60	–	SMD **0.37 SD lower** (0.74 lower to 0.01 lower)	⊕○○○ Very low^c, d, h, i^
**Zhu et al. (** **2024** **)—Activities of daily living**											
3	Randomized trials	Very serious^j^	Serious^a^	Not serious	Serious^e^	None	68	58	–	SMD **0.76 SD lower** (1.3 lower to 0.22 lower)	⊕○○○ Very low^a, e, j^
**Xie et al. (** **2025** **)—Activities of daily living**											
20	Randomized trials	Serious^d^	Very serious^a, k^	Not serious	Not serious	None	419	406	–	SMD **0.64 SD lower** (1 lower to 0.28 lower)	⊕○○○ Very low^a, d, k^
**Li et al. (** **2018a** **)—Mobility**											
9	Randomized trials	Serious^d^	Very serious^a, k^	Not serious	Very serious^c, i^	None	146	144	–	SMD **0.4 SD lower** (0.63 lower to 0.16 lower)	⊕○○○ Very low^a, c, d, i, k^
**Ghayour-Najafabadi et al. (** **2019** **)—Mobility**											
8	Randomized trials	Serious^d^	Very serious^a, h^	Not serious	Serious^e^	None	133	136	–	SMD **0.55 SD lower** (0.93 lower to 0.16 lower)	⊕○○○ Very low^a, d, e, h^
**Tung et al. (** **2019** **)—Mobility**											
6	Randomized trials	Serious^d^	Very serious^a, h^	Not serious	Serious^c^	None	75	60	–	SMD **0.66 SD lower** (1.11 lower to 0.21 lower)	⊕○○○ Very low^a, c, d, h^
**Vaz et al. (** **2019** **)—Mobility**											
6	Randomized trials	Not serious	Very serious^a, h^	Not serious	Not serious	None	94	90	–	SMD **0.97 SD lower** (1.28 lower to 0.66 lower)	⊕⊕○○ Low^a, h^
**Kang et al. (** **2020** **)—Mobility**											
9	Randomized trials	Very serious^g^	Very serious^a, h^	Not serious	Serious^e^	None	146	134	–	SMD **0.48 SD lower** (0.76 lower to 0.19 lower)	⊕○○○ Very low^a, e, g, h^
**Krogh et al. (** **2022** **)—Mobility**											
9	Randomized trials	Not serious	Very serious^a, h^	Not serious	Very serious^c, f^	None	134	117	–	SMD **0.2 SD lower** (0.45 lower to 0.05 higher)	⊕○○○ Very low^a, c, f, h^
**Hofmeijer et al. (** **2023** **)—Mobility**											
4	Randomized trials	Serious^d^	Very serious^a, h^	Not serious	Very serious^c, f^	None	56	56	–	SMD **0.68 SD lower** (1.38 lower to 0.02 higher)	⊕○○○ Very low^a, c, d, f, h^
**Zhou et al. (** **2023** **)—Mobility**											
48	Randomized trials	Serious^d^	Very serious^a, h^	Not serious	Serious^i^	None	317	300	–	SMD **0.35 SD lower** (0.45 lower to 0.24 lower)	⊕○○○ Very low^a, d, h, i^
**Chen et al. (** **2024** **)—Mobility**											
11	Randomized trials	Serious^d^	Serious^h^	Not serious	Very serious^c, f^	None	169	169	–	SMD **0.36 SD lower** (0.85 lower to 0.12 higher)	⊕○○○ Very low^c, d, f, h^
**Jiang et al. (** **2024** **)—Mobility**											
3	Randomized trials	Not serious	Very serious^a, h^	Not serious	Very serious^c, f^	None	42	42	–	SMD **0.6 SD lower** (1.32 lower to 0.1 higher)	⊕○○○ Very low^a, c, f, h^
**Wang J. et al. (** **2024** **)—Mobility**											
12	Randomized trials	Serious^d^	Very serious^a, c^	Not serious	Not serious	None	270	260	–	SMD **0.65 SD lower** (0.98 lower to 0.32 lower)	⊕○○○ Very low^a, c, d^
**Zeng et al. (** **2024** **)—Mobility**											
8	Randomized trials	Not serious	Very serious^a, h^	Not serious	Serious^c^	None	184	173	–	SMD **0.86 SD lower** (1.34 lower to 0.38 lower)	⊕○○○ Very low^a, c, h^
**Wang et al. (** **2025** **)—Mobility**											
10	Randomized trials	Serious^d^	Very serious^a, c^	Not serious	Very serious^c, i^	None	164	139	–	SMD **0.29 SD lower** (0.52 lower to 0.05 lower)	⊕○○○ Very low^a, c, d, i^
**Jia et al. (** **2025** **)—Mobility**											
7	Randomized trials	Not serious	Very serious^a, h^	Not serious	Very serious^c, f^	None	72	58	–	SMD **0.28 SD lower** (0.78 lower to 0.21 higher)	⊕○○○ Very low^a, c, f, h^
**Graef et al. (** **2016** **)—Upper limb activity**											
12	Randomized trials	Serious^d^	Very serious^a, h^	Not serious	Very serious^c, f^	None	185	141	–	SMD **0.06 SD lower** (0.41 lower to 0.29 higher)	⊕○○○ Very low^a, c, d, f, h^
**Zhang et al. (** **2017a** **)—Upper limb activity**											
9	Randomized trials	Serious^j^	Serious^h^	Not serious	Very serious^e, i, l^	None	118	114	–	SMD **0.32 SD lower** (0.55 lower to 0.09 lower)	⊕○○○ Very low^e, h, i, j, l^
**O'Brien et al. (** **2018** **)—Upper limb activity**											
10	Randomized trials	Not serious	Serious^a^	Not serious	Very serious^f, m^	None	109	102	–	SMD **0.46 SD lower** (0 to 0.92 lower)	⊕○○○ Very low^a, f, m^
**van Lieshout et al. (** **2019** **)—Upper limb activity**											
20	Randomized trials	Serious^d^	Very serious^a, h^	Not serious	Serious^f, l^	None	260	235	–	SMD **0.17 SD lower** (0.44 lower to 0.09 higher)	⊕○○○ Very low^a, d, f, h, l^
**Reis et al. (** **2021** **)—Upper limb activity**											
5	Randomized trials	Not serious	Serious^h^	Not serious	Very serious^e, f^	None	83	82	–	SMD **0.03 SD lower** (0.33 lower to 0.28 higher)	⊕○○○ Very low^e, f, h^
**Xi et al. (** **2023** **)—Upper limb activity**											
3	Randomized trials	Serious^d^	Very serious^a, h^	Not serious	Very serious^e, f^	None	24	30	–	SMD **0.34 SD lower** (0.88 lower to 0.2 higher)	⊕○○○ Very low^a, d, e, f, h^
**Jiang et al. (** **2024** **)—Upper limb activity**											
11	Randomized trials	Not serious	Very serious^a, h^	Not serious	Very serious^c, f^	None	123	119	–	SMD **0 SD** (0.02 lower to 0.02 higher)	⊕○○○ Very low^a, c, f, h^
**Zhang J. J. et al. (2024** **)—Upper limb activity**											
19	Randomized trials	Serious^d^	Very serious^a, h^	Serious^n^	Not serious	None	264	223	–	SMD **0.5 SD lower** (0.73 lower to 0.27 lower)	⊕○○○ Very low^a, d, h, n^

CI, confidence interval; SMD, standardized mean difference.

^a^There is a high variability between the rTMS protocols in included studies.

^b^Control groups of the some included studies comprised medications intake.

^c^The sample size of the present meta-analysis was smaller than the required.

^d^The meta-analysis study was classified as “moderate” according to the AMSTAR assessment.

^e^The sample size of the present meta-analysis was smaller than the required.

^f^Imprecise due to the diamond touches the null line.

^g^The meta-analysis study was classified as “critically low” according to the AMSTAR assessment.

^h^There is a critical difference in time since stroke of populations included in different studies.

^i^The meta-analysis effect size did not cross the mCID cut-off.

^j^The meta-analysis study was classified as “low” according to the AMSTAR assessment.

^k^There is a critical difference in time since stroke of populations included in different studies.

^l^There is a critical difference between effect sizes of studies pooled in this forest plot.

^m^There is no available a meta-analysis graph in the study.

^n^Control groups of the some included studies comprised other neuromodulation approaches.

**Table 4a:** Summary of findings (SoF) and certainty of evidence regarding included studies that investigated the effects of transcranial direct current stimulation (tDCS) in ICF body structure and function domains.

**Certainty assessment**							**No. of patients**		**Effect**		**Certainty**

**No. of studies**	**Study design**	**Risk of bias**	**Inconsistency**	**Indirectness**	**Imprecision**	**Other considerations**	**tDCS**	**Sham tDCS**	**Relative (95% CI)**	**Absolute (95% CI)**	
**Elsner et al. (** **2016** **)—Motor function**											
5	Randomized trials	Not serious	Very serious^a, b^	Not serious	Very serious^c, d^	None	183	132	–	SMD **0.36 SD lower** (0.94 lower to 0.21 higher)	⊕○○○ Very low^a, b, c, d^
**Tedesco Triccas et al. (** **2016** **)—Motor function**											
7	Randomized trials	Serious^e^	Very serious^a, b^	Not serious	Extremely Serious^c, d^	None	129	90	–	SMD **0.11 SD lower** (0.38 lower to 0.17 higher)	⊕○○○ Very low^a, b, c, d, e^
**Li et al. (** **2018b** **)—Motor function**											
4	Randomized trials	Serious^e^	Very serious^a, b^	Not serious	Serious^d^	None	49	35	–	SMD **1.54 SD lower** (2.78 lower to 0.29 lower)	⊕○○○ Very low^a, b, d, e^
**Elsner et al. (** **2020** **)—Motor function**											
24	Randomized trials	Not serious	Serious^a^	Not serious	Very serious^c, f^	None	459	333	–	SMD **0.17 SD lower** (0.38 lower to 0.05 higher)	⊕○○○ Very low^a, c, f^
**>Comino-Suárez et al. (** **2021** **)—Motor function**											
10	Randomized trials	Not serious	Very serious^a, b, g^	Not serious	Very serious^c, d^	None	186	156	–	SMD **0.05 SD lower** (0.16 lower to 0.27 higher)	⊕○○○ Very low^a, b, c, d, g^
**Sun et al. (** **2021** **)—Motor function**											
7	Randomized trials	Very serious^h^	Very serious^a, b, g^	Not serious	Very serious^d, i^	None	74	73	–	SMD **0.47 SD lower** (0.78 lower to 0.16 lower)	⊕○○○ Very low^a, b, d, g, h, i^
**Van Hoornweder et al. (** **2021** **)—Motor function**											
22	Randomized trials	Not serious	Very serious^a, b^	Not serious	Very serious^i, j, k^	None	256	257	–	SMD **0.64 SD lower** (0.99 lower to 0.29 lower)	⊕○○○ Very low^a, b, i, j, k^
**Huang et al. (** **2022** **)—motor function**											
12	Randomized trials	Not serious	Very serious^a, b, g^	Not serious	Serious^i, k^	None	458	346	–	SMD **0.83 SD lower** (1.25 lower to 0.4 lower)	⊕○○○ Very low^a, b, g, i, k^
**Ahmed et al. (** **2023** **)—Motor function**											
8	Randomized trials	Very serious^h^	Very serious^a, b^	Not serious	Serious^f, l^	None	151	156	–	SMD **0.34 SD lower** (0.91 lower to 0.24 higher)	⊕○○○ Very low^a, b, f, h, l^
**Lima et al. (** **2023** **)—Motor function**											
6	Randomized trials	Not serious	Very serious^a, b^	Not serious	Very serious^c, d^	None	123	123	–	SMD **0.36 SD lower** (0.9 lower to 0.18 higher)	⊕○○○ Very low^a, b, c, d^
**Lima et al. (** **2024** **)—Motor function**											
9	Randomized trials	Not serious	Very serious^a, b^	Not serious	Very serious^c, d^	None	146	135	–	SMD **0.07 SD lower** (0.31 lower to 0.16 higher)	⊕○○○ Very low^a, b, c, d^
**Ren et al. (** **2024** **)**											
4	Randomized trials	Serious^e^	Serious^b^	Not serious	Very serious^c, d^	None	53	49	–	SMD **0.24 SD lower** (0.63 lower to 0.15 higher)	⊕○○○ Very low^b, c, d, e^
**Tang et al. (** **2024** **)—Motor function**											
42	Randomized trials	Not serious	Very serious^a, b, g^	Not serious	Very serious^i, k, m^	None	807	789	–	SMD **0.22 SD lower** (0.32 lower to 0.12 lower)	⊕○○○ Very low^a, b, g, i, k, m^
**Yu et al. (** **2025** **)—Motor function**											
9	Randomized trials	Very serious^h^	Serious^b^	Not serious	Serious^j^	None	388	399	–	SMD **0.47 SD lower** (0.07 lower to 0.86 lower)	⊕○○○ Very low^b, h, j^

CI, confidence interval; SMD, standardized mean difference.

^a^There is a high variability between the tDCS protocols in included studies.

^b^There is a critical difference in time since stroke of populations included in different studies.

^c^Imprecise due to the diamond touches the null line.

^d^The sample size of the present meta-analysis was smaller than the required.

^e^The meta-analysis study was classified as “moderate quality” according AMSTAR assessment.

^f^The meta-analysis presented a high variability in effect size among the included studies.

^g^Difference between outcomes (in duration of follow-ups).

^h^The meta-analysis study was classified as “critically low quality” according AMSTAR assessment.

^i^Variation between effect sizes of studies.

^j^The meta-analysis effect size did not cross the mCID cut-off.

^k^Not all confidence intervals overlap.

^l^Confidence interval crossing in the minimally important difference.

^m^Imprecise due to confidence intervals included potential for important harm or benefit.

**Table 4b:** Summary of findings (SoF) and certainty of evidence regarding included studies that investigated the effects of transcranial direct current stimulation (tDCS) in ICF activity and participation domains.

**Certainty assessment**							**No. of patients**		**Effect**		**Certainty**

**No. of studies**	**Study design**	**Risk of bias**	**Inconsistency**	**Indirectness**	**Imprecision**	**Other considerations**	**tDCS**	**Sham tDCS**	**Relative (95% CI)**	**Absolute (95% CI)**	
**Tedesco Triccas et al. (** **2016** **)—Activities of daily living**											
5	Randomized trials	Serious^a^	Very serious^b, c^	Not serious	Very serious^d, e^	None	129	71	–	SMD **0.19 SD lower** (0.5 lower to 0.12 higher)	⊕○○○ Very low^a, b, c, d, e^
**Elsner et al. (** **2020** **)—Activities of daily living**											
19	Randomized trials	Not serious	Serious^b, c^	Not serious	Serious^f^	None	412	274	–	SMD **0.28 SD lower** (0.44 lower to 0.13 lower)	⊕⊕○○ Low^b, c, f^
**Comino-Suárez et al. (** **2021** **)—Activities of daily living**											
3	Randomized trials	Not serious	Very serious^b, c^	Not serious	Very serious^d, e^	None	91	63	–	SMD **0.18 SD lower** (0.51 lower to 0.15 higher)	⊕○○○ Very low^b, c, d, e^
**Ahmed et al. (** **2023** **)—Activities of daily living**											
3	Randomized trials	Very serious^g^	Very serious^b, c^	Not serious	Not serious	None	70	71	–	SMD **0.87 SD lower** (1.66 lower to 0.08 lower)	⊕○○○ Very low^b, c, g^
**Tang et al. (** **2024** **)—Activities of daily living**											
11	Randomized trials	Not serious	Very serious^b, c^	Not serious	Not serious	None	283	289	–	SMD **0.37 SD lower** (0.53 lower to 0.2 lower)	⊕⊕○○ Low^b, c^
**Zhang J. J. et al. (2024**)**—Activities of daily living**											
2	Randomized trials	Not serious	Not serious	Not serious	Very serious^d, e^	None	40	39	–	SMD **0.29 SD lower** (0.74 lower to 0.15 higher)	⊕⊕○○ Low^d, e^
**Yu et al. (** **2025** **)—Activities of daily living**											
9	Randomized trials	Very serious^g^	Not serious	Not serious	Not serious	None	238	239	–	SMD **0.95 SD lower** (1.15 lower to 0.75 lower)	⊕⊕○○ Low^g^
**Li et al. (** **2018b** **)—Mobility**											
8	Randomized trials	Serious^a^	Serious^b^	Not serious	Very serious^d, e^	None	64	66	–	SMD **0.35 SD lower** (0.7 lower to 0.01 higher)	⊕○○○ Very low^a, b, d, e^
**Vaz et al. (** **2019** **)—Mobility**											
19	Randomized trials	Not serious	Very serious^b, c^	Not serious	Serious^d, e^	None	183	183	–	SMD **0.1 SD lower** (0.31 lower to 0.11 higher)	⊕○○○ Very low^b, c, d, e^
**Elsner et al. (** **2020** **)—Mobility**											
12	Randomized trials	Not serious	Serious^b, c^	Not serious	Very serious^d, f^	None	135	123	–	SMD **0.32 SD lower** (0.63 lower to 0.01 lower)	⊕○○○ Very low^b, c, d, f^
**Kang et al. (** **2020** **)—Mobility**											
9	Randomized trials	Very serious^g^	Serious^c^	Not serious	Very serious^d, e^	None	132	132	–	SMD **0.29 SD lower** (0.61 lower to 0.02 higher)	⊕○○○ Very low^c, d, e, g^
**Tien et al. (** **2020** **)—Mobility**											
28	Randomized trials	Serious^a^	Serious^h^	Not serious	Serious^f^	None	400	395	–	SMD **0.2 SD lower** (0.34 lower to 0.05 lower)	⊕○○○ Very low^a, f, h^
**Lima et al. (** **2023** **)—Mobility**											
7	Randomized trials	Not serious	Very serious^b, c^	Not serious	Very serious^d, f^	None	73	73	–	SMD **0.41 SD lower** (0.75 lower to 0.08 lower)	⊕○○○ Very low^b, c, d, f^
**Usman et al. (** **2024** **)—Mobility**											
3	Randomized trials	Not serious	Serious^b^	Not serious	Very serious^d, e^	None	65	63	–	SMD **0.25 SD lower** (0.59 lower to 0.1 higher)	⊕○○○ Very low^b, d, e^
**O'Brien et al. (** **2018** **)—Upper limb activity**											
10	Randomized trials	Not serious	Serious^b^	Not serious	Very serious^i^	None	86	81	–	SMD **0.31 SD lower** (0.55 lower to 0.08 lower)	⊕○○○ Very low^b, i^
**Elsner et al. (** **2020** **)—Upper limb activity**											
24	Randomized trials	Not serious	Serious^b, c^	Not serious	Serious^f^	None	459	333	–	SMD **0.31 SD lower** (0.45 lower to 0.16 lower)	⊕⊕○○ Low^b, c, f^
**Comino-Suárez et al. (** **2021** **)—Upper limb activity**											
3	Randomized trials	Not serious	Very serious^b, c^	Not serious	Very serious^d, e^	None	50	84	–	SMD **0.06 SD lower** (0.34 lower to 0.46 higher)	⊕○○○ Very low^b, c, d, e^
**Zhang J. J. et al. (2024** **)—Upper limb activity**											
19	Randomized trials	Serious^a^	Very serious^b, c^	Serious^j^	Not serious	None	264	223	–	SMD **0.65 SD lower** (0.95 lower to 0.36 lower)	⊕○○○ Very low^a, b, c, j^
**Yu et al. (** **2025** **)—Upper limb activity**											
3	Randomized trials	Very serious^g^	Not serious	Not serious	Serious^d^	None	94	96	–	SMD **0.59 SD lower** (0.89 lower to 0.3 lower)	⊕○○○ Very low^d, g^

CI, confidence interval; SMD, standardized mean difference.

^a^The meta-analysis study was classified as “moderate” according to the AMSTAR assessment.

^b^There is a high variability between the tDCS protocols in included studies.

^c^There is a critical difference in time since stroke of populations included in different studies.

^d^The sample size of the present meta-analysis was smaller than the required.

^e^Imprecise due to the diamond touches the null line.

^f^The meta-analysis effect size did not cross the mCID cut-off.

^g^The meta-analysis study was classified as “critically low” according to the AMSTAR assessment.

^h^There is a high variability between the tDCS protocols in included studies.

^i^There is no meta-analysis graph available in the study.

^j^Control group of some studies comprises other approaches of neuromodulation.

For rTMS, the majority of meta-analyses were rated as low or very low certainty of evidence, with the exception of Xiang et al. (2019), which evaluated rTMS effects on activities of daily living (ADL) post-stroke and were rated as having moderate certainty of evidence. For tDCS, all studies demonstrated low or very low certainty of evidence. Many of the meta-analyses showed inconsistencies due to high variability in NIBS protocols and/or imprecision in results, attributed to small effect sizes or broad confidence intervals.

### 3.4 Efficacy of rTMS for body structure/function

**Figure 2:**
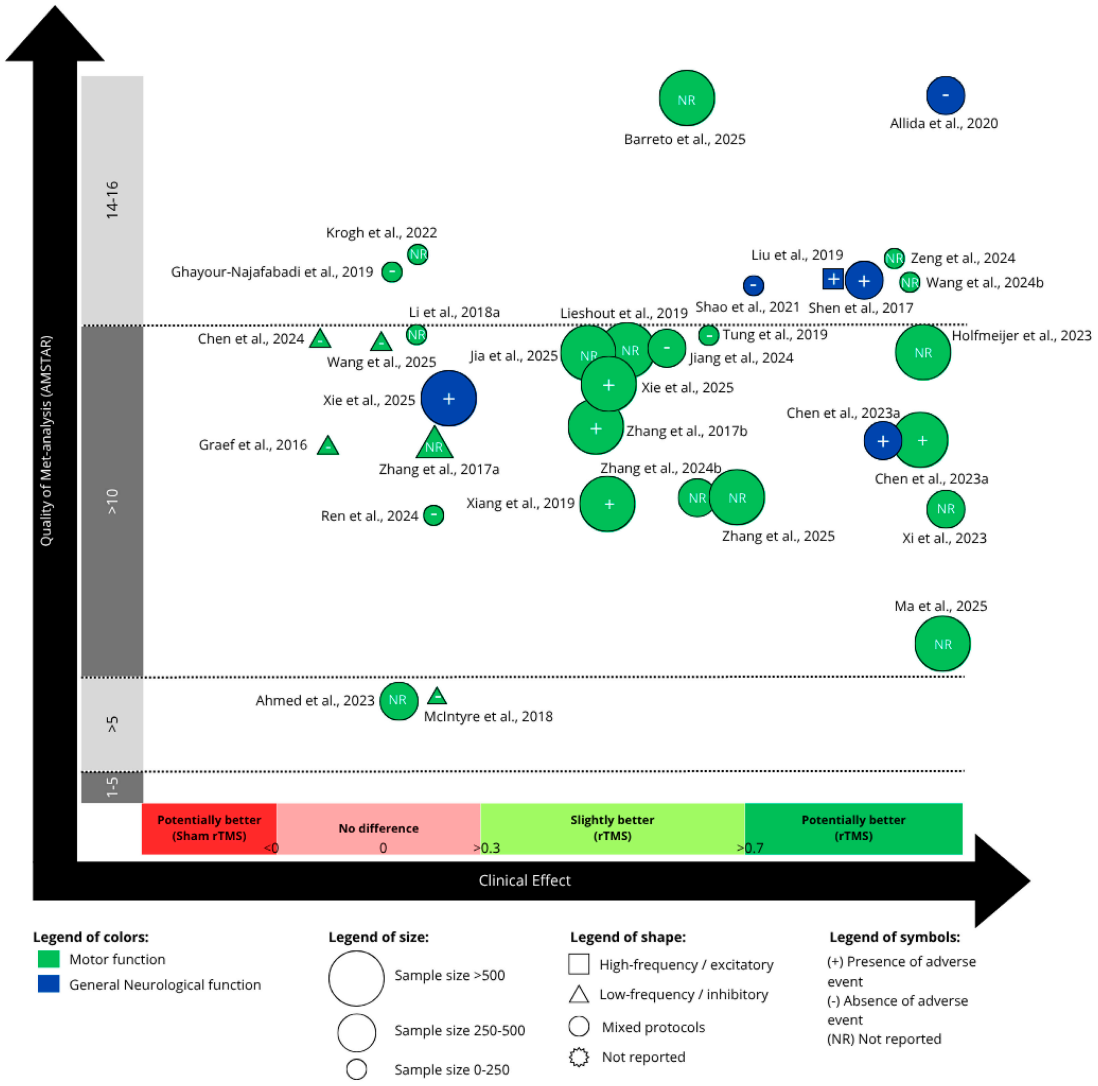
Evidence map for the use of repetitive transcranial magnetic stimulation (rTMS) on body structure and function. “Mixed protocols” indicates that various non-invasive brain stimulation (NIBS) protocols were included within the same meta-analysis.

**Figure 3:**
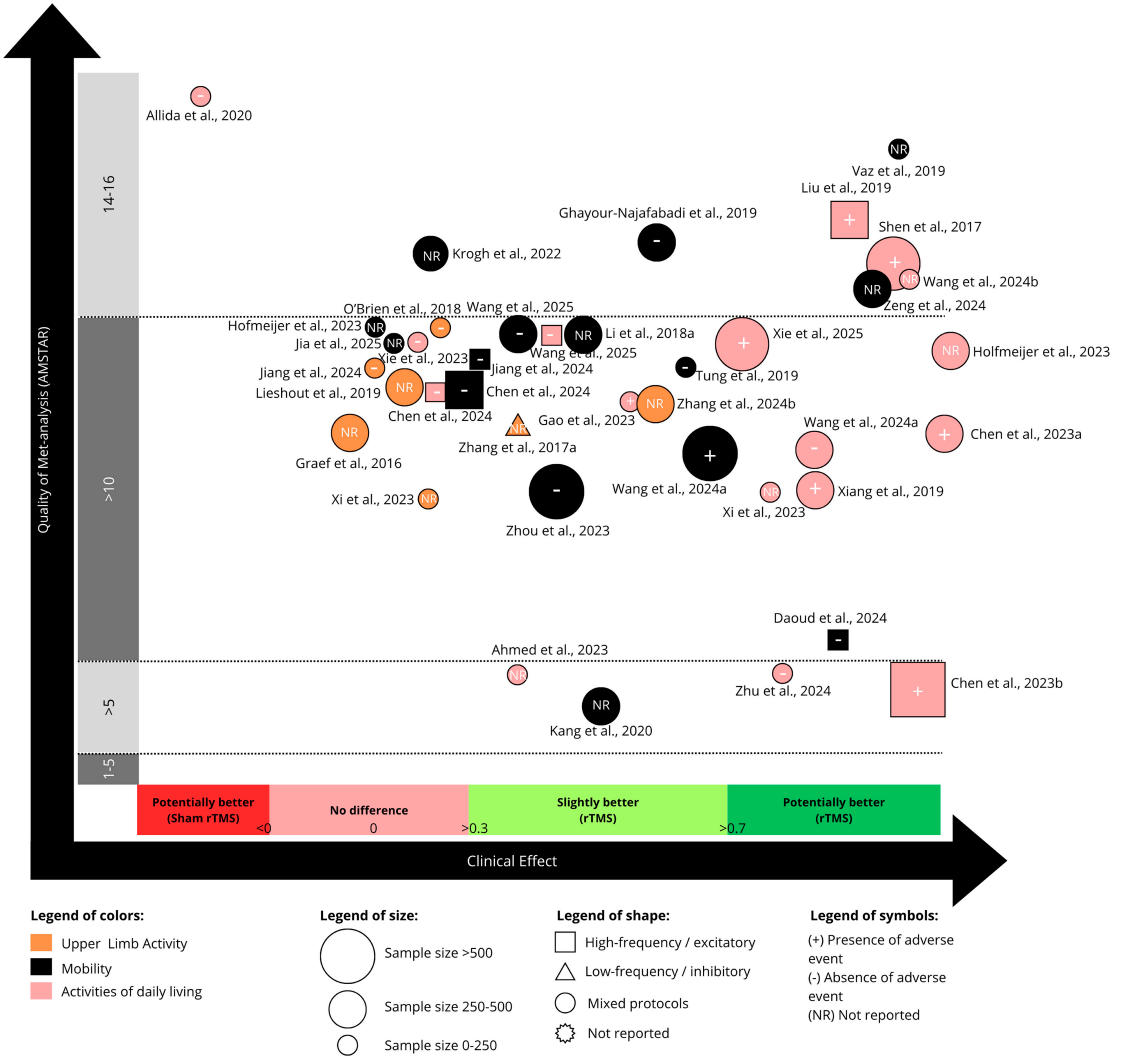
Evidence map for the use of repetitive transcranial magnetic stimulation (rTMS) on activity. “Mixed protocols” indicates that various non-invasive brain stimulation (NIBS) protocols were included within the same meta-analysis.

Figures 2, 3 summarize the clinical efficacy of rTMS across meta-analyses, mapped according to SMD, methodological quality (AMSTAR score), outcome domain (classified according to the ICF framework), sample size, stimulation protocol, and adverse event reporting. Figure 2 presents outcomes related to body structure/function, while Figure 3, discussed in Section 3.5, refers to activity.

Among the 56 included meta-analyses, 32 meta-analyses (57.1%), including between 2 RCT (56 patients; McIntyre et al., 2018) and 36 RCT (1654 patients; Xie et al., 2025), investigated the effect of rTMS on body structure and function domain of the ICF framework, primarily targeting motor function. These studies were categorized into two main outcome domains: motor function (mainly assessed through the Fugl-Meyer Assessment for upper limb- FMA-UL, Fugl-Meyer Assessment for Lower Limb—FMA-LL), and general neurological function (assessed through the National Institutes of Health Stroke Scale—NIHSS).

Of 26 meta-analyses that investigated the effects of rTMS in motor function, 16 (61.5%) observed that rTMS was effective in improving motor function after stroke (Zhang et al., 2017b; Tung et al., 2019; van Lieshout et al., 2019; Xiang et al., 2019; Hofmeijer et al., 2023; Xi et al., 2023; Chen Y. et al., 2023; Jiang et al., 2024; Zhang J. J. et al., 2024; Wang Y. et al., 2024; Zeng et al., 2024; Barreto et al., 2025; Jia et al., 2025; Ma et al., 2025; Xie et al., 2025; Zhang et al., 2025). These studies varied from a moderate (SMD: −0.45; CI: −0.65 to −0.25; Table 3a; Jia et al., 2025) to high (SMD: −1.22; CI: −0.73 to −1.70; Table 3a) effect size (Chen X. et al., 2023; Figure 2). The NNT varied from 8 to 3 (Supplementary Table 2). In general, meta-analyses with larger sample sizes appeared to report results that are more favorable toward rTMS. Notably, the studies by Chen X. et al. (2023), (Xi et al. 2023), (Hofmeijer et al. 2023), and (Ma et al. 2025) reported the largest effect sizes. Five studies were classified as “low quality” of evidence for motor function (Xiang et al., 2019; Xi et al., 2023; Jiang et al., 2024; Barreto et al., 2025; Jia et al., 2025).

Six meta-analyses (100%) reported that general neurological function was slightly (Liu et al., 2019; Allida et al., 2020; Shao et al., 2021; Chen Y. et al., 2023) or potentially meaningful improvements after rTMS treatment (Figure 2). These studies varied from a moderate (SMD: −0.67; CI: −1.02 to −0.32, Table 3a; Shao et al., 2021) to high (SMD: −2.21; CI: −3.32 to −1.09, Table 3a; Liu et al., 2019; Allida et al., 2020) effect size. The NNT varied from 5 to 3 (Supplementary Table 2). However, four of these studies reported high inconsistency and used control groups that included only medication intake, which contributed to their classification as “very low quality of evidence” (Tables 1, 3a). Only two studies were classified as “low quality of evidence” for general neurologic function (Liu et al., 2019; Shao et al., 2021).

### 3.5 Efficacy of rTMS for stroke activity

Thirty-nine meta-analyses, including between 2 studies (128 patients; Ahmed et al., 2023) and 20 studies (825 patients; Xie et al., 2025), investigated the effect of rTMS on outcomes related to ICF domain of activity (Figure 3). The outcomes considered from the studies comprised: (1) upper limb activities; (2) mobility, and (3) ADL. Full details of the outcome measures are available in Table 1.

Of the 39 studies, seven (17.9%) investigated the upper limb activity (Graef et al., 2016; Zhang et al., 2017b; O'Brien et al., 2018; van Lieshout et al., 2019; Chen X. et al., 2023; Xi et al., 2023; Jiang et al., 2024; Zhang J. J. et al., 2024), 18 studies (46.2%) investigated performance in ADL (Liu et al., 2019; Xiang et al., 2019; Allida et al., 2020; Ahmed et al., 2023; Gao et al., 2023; Hofmeijer et al., 2023; Chen Y. et al., 2023; Xie et al., 2023, 2025; Xi et al., 2023; Chen et al., 2024; Daoud et al., 2024; Jiang et al., 2024; Ren et al., 2024; Wang J. et al., 2024; Wang Y. et al., 2024; Zhu et al., 2024; Wang et al., 2025) and 14 (35.9%) focused on mobility (Figure 3; Li et al., 2018a; Ghayour-Najafabadi et al., 2019; Tung et al., 2019; Vaz et al., 2019; Kang et al., 2020; Krogh et al., 2022; Hofmeijer et al., 2023; Zhou et al., 2023; Chen et al., 2024; Jiang et al., 2024; Wang J. et al., 2024; Zeng et al., 2024; Wang et al., 2025; Jia et al., 2025). Of the seven studies on upper limb activity, only two (28.6%) reported that rTMS was effective in improving this outcome after stroke. These studies showed a low (SMD: −0.32; CI: −0.55 to −0.09; Zhang et al., 2017a) to moderate (SMD: −0.50; CI: −0.73 to −0.27) effect size, with low heterogeneity index (34.2%; *p*-value 0.07; Table 1; Zhang N. et al., 2024). The NNT varied from 6 to 13 (Supplementary Table 2). However, all studies that investigated the effects of rTMS in upper limb activity showed considerable variability in intervention protocols and lower sample sizes and a large CI (Table 3b), leading to an overall very low quality of evidence.

Of the 14 studies that investigated the rTMS effects on mobility, seven (50 %) studies (Li et al., 2018a; Ghayour-Najafabadi et al., 2019; Tung et al., 2019; Kang et al., 2020; Zhou et al., 2023; Wang J. et al., 2024; Wang et al., 2025) found that rTMS slightly and three (21.4%) potentially improved mobility outcomes (Figure 3; Vaz et al., 2019; Daoud et al., 2024; Zeng et al., 2024). These studies showed a low SMD: −0.29 (−0.52 to −0.05; Wang et al., 2025) to high (SMD: −0.97; CI: −1.28 to −0.66; Vaz et al., 2019) effect size, with low inconsistency indices (Table 1). The NNT varied from 4 to 13 (Supplementary Table 2). The study with higher effect size was classified as “low quality of evidence” due to substantial variation in rTMS protocols and heterogeneity in participant characteristics, particularly regarding time since stroke (Table 3b).

Finally, 15 (83.3%) of 18 studies reported that performance in ADL was potentially or slightly improved after rTMS in stroke survivors (Figure 3; Shen et al., 2017; Liu et al., 2019; Xiang et al., 2019; Ahmed et al., 2023; Chen X. et al., 2023; Chen Y. et al., 2023; Gao et al., 2023; Hofmeijer et al., 2023; Xi et al., 2023; Daoud et al., 2024; Ren et al., 2024; Zhu et al., 2024; Wang Y. et al., 2024; Xie et al., 2025; Wang et al., 2025). One study was classified as moderate quality of evidence (Xiang et al., 2019). Besides the high effect size (SMD: −0.82; IC: −1.05 to −0.59; NNT: 5) with low heterogeneity index (0%, *p*-value: 0.78), we downgrade one point in risk of bias, because this study was classified as “moderate” in AMSTAR classification (Table 3b). Three studies (30%) were classified as “low quality of evidence” (Chen Y. et al., 2023; Shen et al., 2017; Liu et al., 2019), besides they presented high effect sizes (Supplementary Table 2, Table 3b) and 14 (77.8%) studies were classified as “very low quality of evidence.”

### 3.6 Efficacy of tDCS for body structure/function

**Figure 4:**
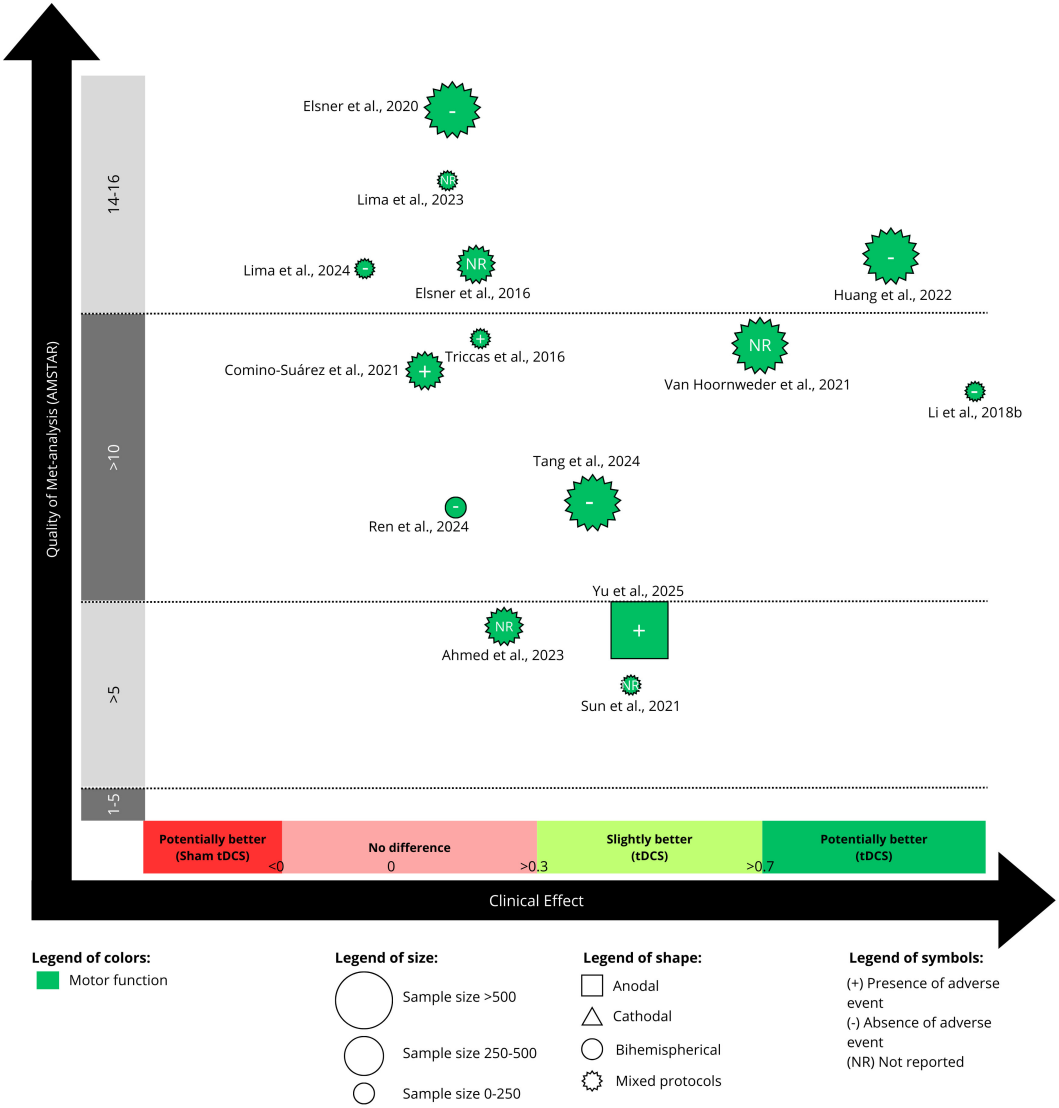
Evidence map for the use of transcranial direct current stimulation (tDCS) on body structure and function. “Mixed protocols” indicates that various non-invasive brain stimulation (NIBS) protocols were included within the same meta-analysis.

**Figure 5:**
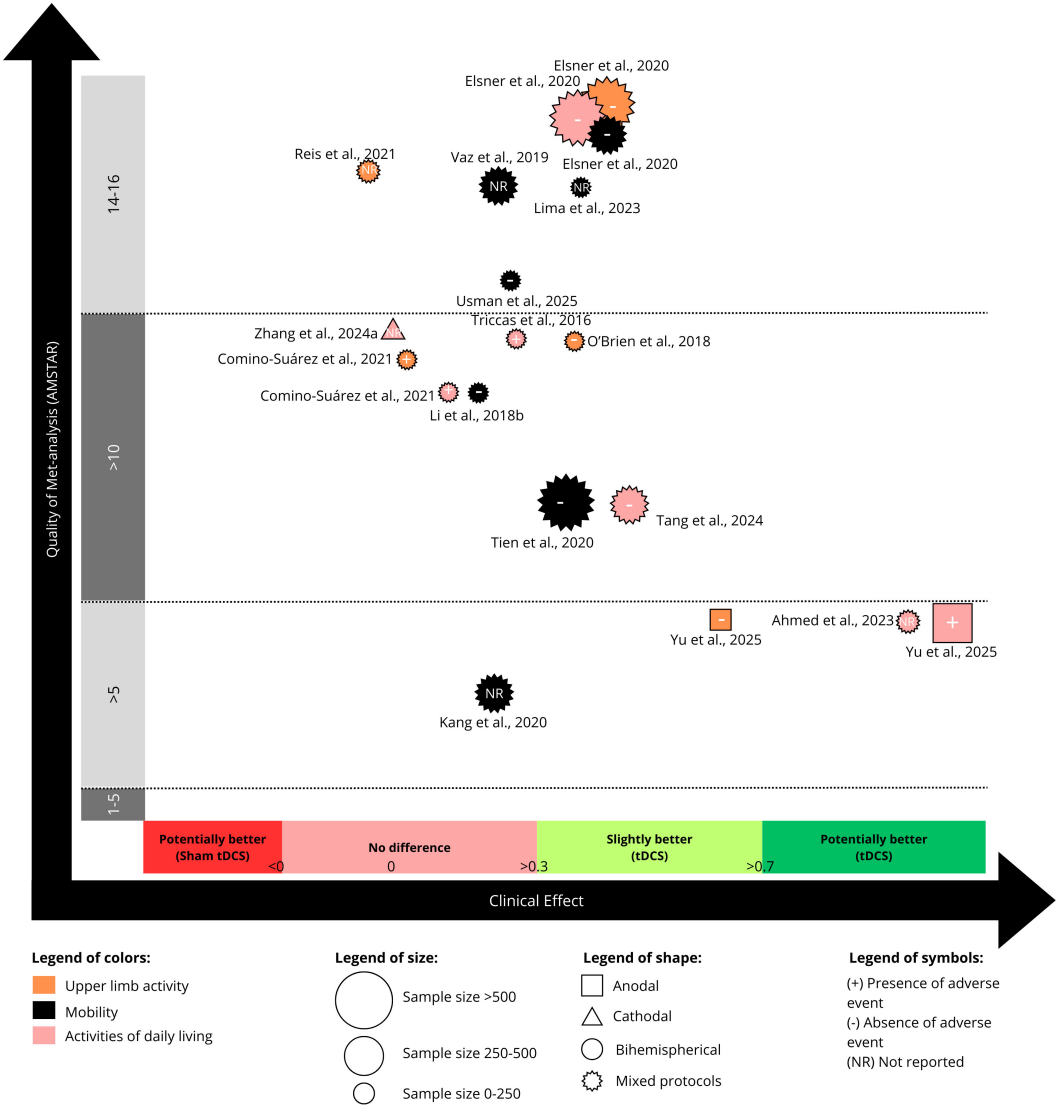
Evidence map for the use of transcranial direct current stimulation (tDCS) on activity. “Mixed protocols” indicates that various non-invasive brain stimulation (NIBS) protocols were included within the same meta-analysis.

Figures 4, 5 summarize the clinical efficacy of tDCS across meta-analyses, mapped according to SMD, methodological quality (AMSTAR score), outcome domain (classified according to the ICF framework), sample size, stimulation protocol, and adverse event reporting. Figure 4 presents outcomes related to body structure/function, while Figure 5, discussed in Section 3.6, refers to activity.

Fourteen meta-analyses, ranging from 4 studies (84 patients; Li et al., 2018b) to 42 studies (1596 patients; Tang et al., 2024), investigated the effect of tDCS on body structure and function. The outcome measures considered were: (1) FMA-UE, (2) FMA-LE, (3) MAS. Details on each measure are provided in Table 1.

Six of fourteen (42.9%) reported that tDCS was slightly or potentially effective in improving motor function after stroke (Figure 4; Li et al., 2018b; Sun et al., 2021; Van Hoornweder et al., 2021; Huang et al., 2022; Tang et al., 2024; Yu et al., 2025). These studies reported effect sizes ranging from low (SMD: −0.22; CI: −0.32 to −0.12; Tang et al., 2024) to high (SMD: −1.54; 95% CI: −2.78 to −0.29; Li et al., 2018b). The NNT varied from 3 to 17, with very serious inconsistency and imprecision issues, with higher heterogeneity indexes (*I*^2^ > 40%; *p*-value < 0.01; Table 1). Thus, the overall quality of evidence for this outcome was deemed “very low” (Table 4a).

### 3.7 Efficacy of tDCS for stroke activity

Nineteen meta-analyses investigated the effect of tDCS on outcomes related to ICF domain of activity. Of the 19 studies, five (26.3%) examined upper limb activity (O'Brien et al., 2018; Elsner et al., 2020; Comino-Suárez et al., 2021; Reis et al., 2021; Yu et al., 2025), seven (36.8%) investigated mobility (Li et al., 2018b; Vaz et al., 2019; Elsner et al., 2020; Kang et al., 2020; Tien et al., 2020; Lima et al., 2023; Usman et al., 2024), and seven (33.3%) focused on ADL post-stroke (Tedesco Triccas et al., 2016; Elsner et al., 2020; Comino-Suárez et al., 2021; Ahmed et al., 2023; Zhang N. et al., 2024; Tang et al., 2024; Yu et al., 2025; Figure 5).

Three studies of five (60%) observed that tDCS was effective in improving upper limb activity. Reported effect sizes were low (SMD: −0.31; CI: −0.55 to −0.01 and SMD: −0.31; CI: −0.45 to −0.16; NNT: 12) or moderate (SMD: -0.59; CI: -0.89 to -0.30; NNT: 7), with low heterogeneity indices (O'Brien et al., 2018; Elsner et al., 2020; Yu et al., 2025) (Table 1). However, the studies exhibited considerable variability in the included protocols and the time since stroke onset, then we downgraded one point in inconsistency. One study did not present a meta-analysis graph (O'Brien et al., 2018), because of this, we downgraded two points for imprecision (for details, see the Table 4b).

Regarding mobility, three (Elsner et al., 2020; Tien et al., 2020; Lima et al., 2023) of seven studies (42.9%) reported slight improvements following tDCS (Figure 5). These studies showed a low effect size with low inconsistency indices. The NNT varied from 9 to 18. Overall, the studies were classified as “very low quality of evidence” because it presented high variability between tDCS protocols, included individuals with different times since stroke, presented a small sample size and larger CI intervals (Table 4b, Supplementary Table 2).

Finally, four of seven studies (57.1%) showed that the performance in ADL was slightly (Elsner et al., 2020; Tang et al., 2024) or potentially (Ahmed et al., 2023; Yu et al., 2025) increased following tDCS. The NNT varied from 5 to 13. The study with higher effect size was classified as “very low quality of evidence” because it was classified as “critically low” in AMSTAR, a high variability between tDCS protocols and included patients with different time since stroke (Table 4b).

**Figure 6:**
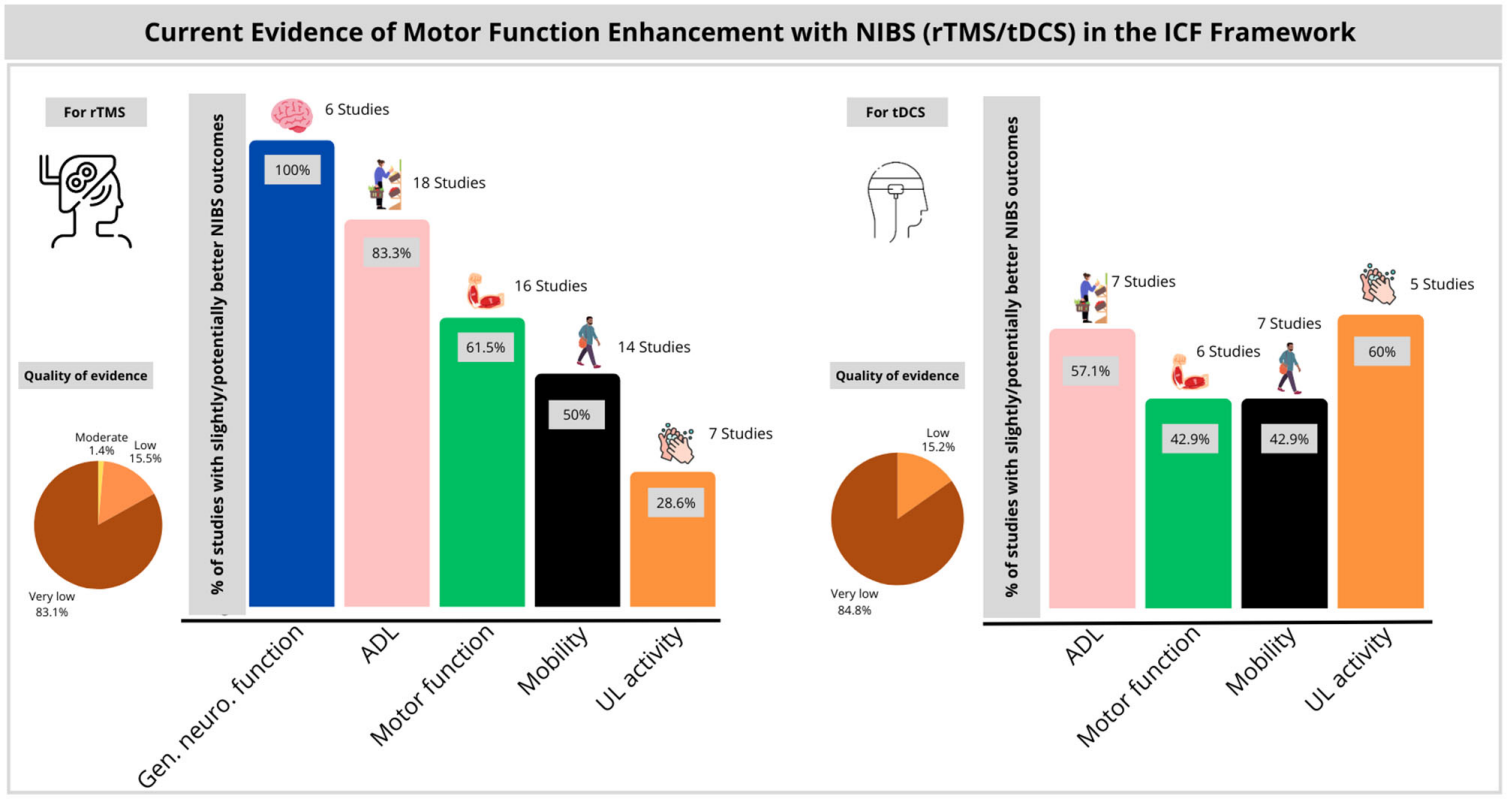
A summary of the current evidence supporting the effects of repetitive transcranial magnetic stimulation (rTMS) and transcranial direct current stimulation (tDCS) within the ICF framework in post-stroke individuals.

We summarized the current evidence supporting motor function improvements with NIBS (rTMS/tDCS) within the principal domains of the ICF framework in Figure 6. This figure presents a visual synthesis of the motor functions most frequently reported as positively influenced by NIBS, accompanied by a rank-ordered list indicating the number and percentage of studies supporting each functional outcome.

### 3.8 Safety of NIBS for stroke

Table 1 also reports the adverse events reported in the rTMS and tDCS studies. 10 (25.6%) meta-analyses of rTMS (Shen et al., 2017; Zhang et al., 2017b; Liu et al., 2019; Tung et al., 2019; Xiang et al., 2019; Gao et al., 2023; Chen X. et al., 2023; Chen Y. et al., 2023; Wang J. et al., 2024; Xie et al., 2025) and three (14.3%) of tDCS report the occurrence of severe adverse effects after the stimulation (Comino-Suárez et al., 2021; Tedesco Triccas et al., 2016; Yu et al., 2025). For rTMS, commonly reported adverse effects include: headache, gastrointestinal reaction, tinnitus, feel weak, anxiety, nausea, tingling, dizziness, fatigue, drowsiness, neck pain, cast irritation, palpitation, and neurocardiogenic syncope. For tDCS, commonly reported adverse effects included: headache, dizziness, fatigue and tingling (Table 1). It is also important to highlight that some meta-analysis failed to report adverse effects in the results: thirteen for rTMS (37.1% of rTMS studies; Zhang et al., 2017a; Li et al., 2018a; van Lieshout et al., 2019; Krogh et al., 2022; Hofmeijer et al., 2023; Xi et al., 2023; Wang Y. et al., 2024; Zeng et al., 2024; Zhang N. et al., 2024; Barreto et al., 2025; Jia et al., 2025; Ma et al., 2025; Zhang et al., 2025), six for tDCS (37.5% of tDCS studies; Elsner et al., 2016; Reis et al., 2021; Sun et al., 2021; Van Hoornweder et al., 2021; Lima et al., 2023; Zhang J. J. et al., 2024), and four for rTMS and tDCS studies (80% of included meta-analyses; O'Brien et al., 2018; Vaz et al., 2019; Kang et al., 2020; Ahmed et al., 2023).

## 4 Discussion

This umbrella review is the first to synthesize and assess the quality of evidence from meta-analyses on NIBS in stroke rehabilitation, using the principal domains of ICF as a framework. Regarding the body structure/function domain, rTMS was more often associated with moderate to high effect sizes, particularly for general neurological function. In contrast, although some meta-analyses suggested that tDCS may have slight to potentially meaningful effects on motor function recovery, the certainty of this evidence was rated as very low due to serious concerns related to heterogeneity, imprecision, and variability in stimulation protocols. In the activity domain, both techniques showed modest effects, with rTMS demonstrating more favorable results for ADL than for mobility or upper limb activity. tDCS effects on activity-related outcomes were generally limited and supported by low to very low certainty of evidence across most outcomes. Furthermore, although no serious adverse events were reported across the meta-analyses, moderate adverse effects, including headache, fatigue, and occasional episodes of neurocardiogenic syncope, were documented. These findings indicate that while NIBS appears to have an acceptable safety profile, its tolerability may vary among individuals and should be carefully monitored in clinical applications.

When interpreting the magnitude of treatment effects observed in this umbrella review, it is important to consider thresholds for clinical relevance (Citrome, 2014). Although there is no universally accepted cutoff, we adopted a standardized mean difference (SMD) of 0.7 as a conservative benchmark for clinically meaningful effects, which is slightly above the conventional threshold for a moderate effect size (Rahlfs and Zimmermann, 2019; Zieliński, 2025). Effects at or above this level may reflect changes likely to translate into noticeable improvements in patient outcomes. However, it is important to recognize that the clinical significance of these effects can vary depending on the specific outcome assessed, the patient population, and the context of rehabilitation (Cuijpers et al., 2014). Therefore, while effect sizes below this threshold should be interpreted with caution, clinical decision-making should also integrate factors such as feasibility, patient preferences, and safety (Page, 2014). Indeed, we found more consistent clinically meaningful effects of rTMS in improving motor function, general neurologic function and performance in ADL. For tDCS, the body of evidence remain uncertain, with few studies presenting clinically meaningful effects just for motor function and performance in ADL.

### 4.1 Methodological quality of meta-analyses

The methodological quality of the included meta-analyses, as assessed by the AMSTAR tool, was predominantly moderate to high, with over 80% of the studies meeting key methodological criteria. These findings suggest increasing adherence to rigorous review practices and a growing methodological maturity in this research area. Only a small proportion were rated as low or critically low quality (14.6%), suggesting that some methodological inconsistencies still remain.

There were some limitations regarding the quality of evidence of the studies included in this Umbrella Review, such as the variability of protocols, which limited comparisons between studies, and the relatively small sample sizes that may compromise statistical robustness and reduce the generalizability of the results. Furthermore, the predominance of “low” or “very low” certainty ratings according to GRADE reflects imprecision and inconsistency in effect estimates, largely due to heterogeneity in intervention protocols, risk of bias, and variability in outcome measures and evaluation methods. These factors combined reduce confidence in the reliability of the findings and underscore the need for more rigorous, standardized randomized controlled trials and meta-analyses to strengthen the evidence base and improve the clinical applicability of NIBS in stroke rehabilitation.

The most frequent sources of methodological concern were the lack of justification for deviations from the original review protocols, incomplete or insufficiently reported search strategies, and the omission of funding information for the primary studies. These issues have important implications for the interpretation of findings. Unreported protocol deviations reduce transparency and increase the risk of selective reporting, which may introduce bias in the synthesis process. Inadequate search strategies can result in the exclusion of relevant studies, particularly negative trials, potentially inflating the estimated effects due to publication bias. Moreover, the failure to report funding sources of included studies limits the ability to assess conflicts of interest, which could compromise the neutrality of the evidence base. To address these issues, future reviews should aim to ensure compliance with all items outlined in the PRISMA 2020 guidelines, which are essential to enhance the credibility, transparency, and reproducibility of evidence syntheses in the field of NIBS.

### 4.2 Effects of rTMS on ICF domains in post-stroke rehabilitation

In our review, rTMS demonstrated the most consistent and clinically relevant effects within the ICF domain of body structure and function, particularly general neurological function. Nearly all studies evaluating this outcome reported positive effects of rTMS, with relatively low variability in effect magnitude, ranging from moderate (Shao et al., 2021) to high (Allida et al., 2020). Similarly, among the 26 meta-analyses that investigated motor function, more than half reported significant improvements in stroke recovery, with effect sizes also ranging from moderate to high.

Although the overall body of evidence supports a beneficial effect of rTMS in improving outcomes within the ICF domain of body structure and function, the findings related to motor function were more heterogeneous in terms of effect magnitude. This variability likely reflects multiple contributing factors, including differences in stimulation protocols (e.g., frequency, intensity, target site), patient characteristics (e.g., time since stroke, severity), methodological quality, and the number of studies synthesized within each meta-analysis. Meta-analyses with larger sample sizes, such as those by Chen Y. et al. (2023), Xi et al. (2023), and Hofmeijer et al. (2023), tended to report stronger and relevant effect sizes. This observation is consistent with methodological recommendations emphasizing the importance of adequately powered studies to reduce the risk of bias and enhance the precision of effect estimates (Ioannidis, 2005).

It is important to note that, compared with motor function outcomes, the effects of rTMS on general neurological function (e.g., as measured by NIHSS) were more consistent across studies, despite variations in stimulation protocols. One possible explanation for this lower variability is that such outcomes are broader in scope, capturing diffuse neurological changes that may occur across multiple functional systems. In contrast, motor function outcomes, particularly those assessing specific limb performance with tools such as the FMA, are more narrowly focused and may be more susceptible to individual variability, such as lesion location, stroke severity, or rehabilitation context. This discrepancy highlights the importance of carefully selecting and clearly defining outcome measures in neuromodulation trials. Notably, the methodological quality of the studies evaluating general neurological function was also higher (e.g., Allida et al., 2020; Liu et al., 2019; Shen et al., 2017), which may have contributed to more consistent results. Methodological rigor is known to influence the reliability of meta-analytic findings; systematic reviews with high AMSTAR-2 scores are more likely to produce valid and unbiased estimates (Shea et al., 2017).

Among the meta-analyses assessing activity outcomes, the effects of rTMS were generally less consistent and less robust than those observed for body structure and function, except for ADL. Sixteen out of eighteen studies evaluating ADL reported slight to potentially meaningful improvements following rTMS. Among them, only the study by Xiang et al. (2019) achieved moderate certainty of evidence and reported a large effect size with low heterogeneity, likely due to its use of subgroup analyses based on stroke population characteristics and the application of optimized stimulation parameters. In contrast, the evidence for mobility was less consistent. Although the majority of studies reported positive effects, effect sizes varied widely from small to large and all were rated as low or very low certainty of evidence, primarily due to heterogeneity in stimulation protocols and participant characteristics. The weakest evidence was observed for upper limb activity: only two out of seven studies demonstrated a statistically significant effect, and all were rated as very low certainty, largely also reflecting protocol inconsistencies.

While most meta-analyses evaluating rTMS for post-stroke rehabilitation report slight to potentially meaningful effects, especially for outcomes such as general neurological function and ADL, differences in stimulation parameters, patient characteristics, and outcome definitions likely obscure the consistency of the evidence and contribute to the predominance of low or very low certainty ratings in GRADE assessments. In many meta-analyses, the inclusion of trials with markedly divergent methodologies has reduced the consistency and precision of the pooled estimates, ultimately lowering the overall quality of the evidence.

At the same time, the NIBS field is moving toward increasingly personalized rTMS interventions, with growing efforts to tailor stimulation protocols based on lesion location, functional reserve, neurophysiological markers, and time since stroke (Hildesheim et al., 2022). While this individualized approach holds promise for improving patient-level outcomes, it also introduces new layers of heterogeneity that may further complicate evidence synthesis. As protocols become more specific to individual profiles, future meta-analyses may face greater challenges in aggregating results, potentially reinforcing the trend of low certainty of evidence unless new strategies are developed to standardize personalization frameworks without compromising clinical relevance. Establishing clinical guidelines that balance inter-individual variability with methodological rigor will be crucial.

When comparing the domains of body structure/function and activity, rTMS appears to have a stronger and more consistent effect on body structure and function outcomes than on activity outcomes. As discussed earlier, most meta-analyses evaluating motor and general neurological function reported moderate to high effect sizes with relatively low variability. In contrast, outcomes related to activity, particularly those assessing mobility and upper limb use, demonstrated greater heterogeneity and lower certainty of evidence. One possible explanation is that improvements in impairment-level outcomes (e.g., motor function) may not directly translate into higher-level functional activities, especially in the absence of structured, context-specific rehabilitation. Functional outcomes such as mobility and upper limb use often require meaningful behavior change, including the integration of newly recovered abilities into daily routines. Moreover, the relatively short duration of most NIBS protocols—typically limited to 10 to 20 sessions—may be insufficient to promote the sustained engagement and task-specific motor learning needed to drive long-term functional gains in real-world settings.

### 4.3 Effects of tDCS on ICF domains in post-stroke rehabilitation

A substantial number of meta-analyses have examined the effects of tDCS on motor recovery and functional performance after stroke. However, findings across studies remain heterogeneous, particularly for outcomes related to body structure and function. While some reviews reported moderate to large effect sizes for motor improvements (Li et al., 2018b; Sun et al., 2021; Van Hoornweder et al., 2021; Huang et al., 2022), the lack of consistency across meta-analyses and the predominance of low-certainty evidence hinder the formulation of clear clinical recommendations. In contrast, outcomes related to activity, especially ADL, more frequently showed evidence of benefit (O'Brien et al., 2018; Elsner et al., 2020; Tien et al., 2020; Ahmed et al., 2023; Lima et al., 2023; Tang et al., 2024), though with smaller effect sizes. These findings were also characterized by substantial methodological limitations, such as such as the low quality of several meta-analyses, inconsistency in methods of the included studies in each meta-analyses and imprecise results.

Meta-analyses with larger sample sizes (Huang et al., 2022; Van Hoornweder et al., 2021; Tang et al., 2024; Elsner et al., 2020) tended to report more robust and stable effect estimates, underscoring the pivotal role of sample size in the reliability of pooled outcomes. For instance, while Huang et al. (2022) and Van Hoornweder et al. (2021) reported large standardized mean differences, these were accompanied by very high inconsistency indices (*I*^2^ > 60%). This reinforces a well-recognized concern in complex interventions such as NIBS: small, underpowered studies are more prone to random error and effect size inflation (Button et al., 2013; Mitra et al., 2019; Andrade, 2020). The precision and reliability of effect estimates can be significantly improved by increasing sample size and maintaining methodological rigor.

Methodologically, the body of evidence on tDCS appears less robust than that on rTMS. Most reviews were rated as low or critically low quality, according to the AMSTAR-2 tool, and all but one were classified as providing low or very low certainty of evidence by GRADE. These methodological limitations, such as lack of protocol registration, absence of publication bias assessment, and inconsistencies in risk of bias evaluation, undermine the reliability of the conclusions and underscore the need for higher-quality evidence syntheses in this area (Shea et al., 2017).

Taken together, the findings suggest that although tDCS holds promise for improving motor recovery and functional performance in individuals with stroke, current evidence remains limited by small sample sizes, heterogeneous protocols, and methodological weaknesses. Future research should address these gaps through well-designed, adequately powered trials and rigorous evidence syntheses.

Importantly, the clinical heterogeneity observed across meta-analyses likely reflects, at least in part, the individualized nature of tDCS application. As a neuromodulation technique, tDCS is often tailored to a patient's specific clinical characteristics, such as stroke chronicity, lesion site, or level of impairment (Simonetta-Moreau, 2014; Baltar et al., 2020), resulting in a degree of protocol variability that is not only expected but also necessary to accommodate diverse rehabilitation needs. While this variability complicates direct comparisons and evidence synthesis, it also underscores the importance of developing analytic strategies capable of capturing clinically relevant heterogeneity, rather than penalizing it as a methodological weakness.

Although this overview selected the most representative meta-analyses for each outcome, many incorporated subgroup analyses within their synthesis. While this strategy enhances generalizability, it may also have obscure clinically meaningful effects linked to more individualized stimulation parameters. By aggregating heterogeneous data without stratification, the resulting estimates tend to show greater variability, which may lead to downgraded certainty of evidence and attenuate effect sizes. The absence of subgroup analyses, despite their potential to identify more effective, tailored interventions, may therefore contribute to underestimating the therapeutic potential of tDCS in specific patient profiles. Consequently, the true clinical impact of tDCS may have been partially diminished by fragmented or overly narrow analytical approaches, reinforcing the need for meta-analyses that balance granularity with statistical power.

### 4.4 Safety of NIBS for the stroke treatment

The reporting of adverse effects across the included meta-analyses was limited and inconsistent, restricting the ability to comprehensively assess the safety of NIBS after stroke. Although some studies described mild to moderate side effects—such as headache, dizziness, fatigue, and tingling—severe adverse events were reported in only a minority of meta-analyses (25.6% for rTMS and 14.3% for tDCS). The heterogeneity in types and frequencies of adverse effects likely reflects both real differences across protocols and populations, as well as variability in how primary studies monitor and report safety outcomes. Notably, more than one-third of the meta-analyses failed to mention adverse events at all. This underreporting represents a significant methodological limitation in the NIBS literature and underscores the urgent need for standardized reporting of safety data in future trials and evidence syntheses. Without such transparency, the clinical interpretation of risk–benefit ratios remains incomplete.

### 4.5 Limitations and future perspectives

As an umbrella review, this study plays an important role in promoting broader recognition of NIBS and informing professionals about its potential clinical benefits. However, few limitations must be acknowledged. First, the literature search was conducted exclusively in the MEDLINE (PubMed) database. Although PubMed is a widely recognized and comprehensive source for health-related research, restricting the search to a single database may have limited the retrieval of additional relevant meta-analyses. Additionally, although the search strategy was validated by experts in scientific methodology and NIBS, we did not include a medical librarian in the development of the search terms, which might have further optimized the process. Future updates should consider incorporating databases such as EMBASE, Scopus, and the Cochrane Library to enhance comprehensiveness and reduce publication bias.

Second, due to substantial heterogeneity in outcome measures and reporting, we were unable to provide a clear and exhaustive analysis of outcomes stratified by specific ICF domains and subdomains. This inconsistency—together with frequent overlap across domains—limited our ability to determine whether outcomes referred to walking, transfers, or other specific aspects of mobility. These limitations reflect variability in outcome reporting in the primary studies and meta-analyses. For example, it was not consistently possible to distinguish whether mobility-related outcomes referred specifically to walking, transfers, bed mobility, or community ambulation. Although we acknowledge that this level of detail would enhance the clinical applicability of the findings, the limitation stems from inconsistencies in outcome reporting within the primary studies and meta-analyses synthesized.

This methodological variability also limited the ability to determine which specific configurations might be associated with greater therapeutic efficacy. Similarly, although clinical factors such as lesion location, time since stroke, and lesion extent are known to influence individual responsiveness to NIBS, the available evidence did not allow for a more granular analysis of these variables. Additionally, identifying predictors of treatment response—distinguishing responders from non-responders—would require access to individual participant data or consistent subgroup analyses, which were rarely available across the reviews.

NIBS has evolved significantly in recent years, becoming an increasingly central intervention in post-stroke rehabilitation. The principle of personalization is fundamental to this approach, as it allows protocols to be adapted based on individual clinical characteristics, such as stroke severity, lesion location and patient functional profile (Coêlho et al., 2021). Personalized stimulation involves tailoring parameters, protocols, and patient selection criteria, optimizing treatment effectiveness and improve outcomes in an individualized manner (Kesselheim et al., 2023; Wessel et al., 2024). For this reason, techniques such as neuromodulation may yield suboptimal results when applied with “one size fits all” treatment, the lack of personalization can limit treatment efficacy (Ovadia-Caro et al., 2019). Our umbrella review was not designed to investigate distinctions between specific stimulation protocols or patient characteristics. Future reviews incorporating subgroup analyses should aim to identify stimulation protocols associated with greater therapeutic efficacy, as well as the investigation of whether clinical factors such as lesion location, time since stroke, stimulation dose, and lesion extent can predict responsiveness to NIBS. In addition, more studies with larger samples and long-term follow-up is needed to assess the durability of NIBS effects in post-stroke recovery.

## Data Availability

The original contributions presented in the study are included in the article/Supplementary material, further inquiries can be directed to the corresponding author.
