# Supplementary material for: Non-invasive brain stimulation for stroke-related motor impairment and disability: an umbrella review of systematic review and meta-analysis
Source: Front Neurosci. 2025 Sep 9;19:1633986. doi: 10.3389/fnins.2025.1633986 (PMC12454317; doi:10.3389/fnins.2025.1633986)
Supplement: Supplementary file 1 [file Table_1.docx]

**Table S1.** Search strategy and number of articles retrieved in the umbrella review

| **Search Strategy** | **Number of retrieved reviews** |
| --- | --- |
| "Transcranial Magnetic Stimulation"[Mesh) AND "Stroke"[Mesh] | 137 |
| "TMS" AND "Stroke"[Mesh] | 32 |
| "repetitive Transcranial Magnetic Stimulation" AND "Stroke"[Mesh] | 110 |
| "rTMS" AND "Stroke"[Mesh] | 100 |
| "Noninvasive Brain Stimulation" AND "Stroke"[Mesh] | 28 |
| "transcranial direct current stimulation"[Mesh) AND "Stroke"[Mesh] | 100 |
| "tDCS" AND "Stroke"[Mesh] | 82 |
| "brain polarization" AND "Stroke"[Mesh] | 0 |
| "transcranial alternating current stimulation" AND "Stroke"[Mesh] | 1 |
| "tACS" AND "Stroke"[Mesh] | 5 |
| "transcranial electrical stimulation" AND "Stroke"[Mesh] | 2 |
| "tES" AND "Stroke"[Mesh] | 5 |
| "transcranial random noise stimulation" AND "Stroke"[Mesh] | 0 |
| "tRNS" AND "Stroke"[Mesh] | 0 |
| "transcranial cerebellar direct current stimulation" AND "Stroke"[Mesh] | 0 |
| "cerebellar direct current stimulation" AND "Stroke"[Mesh] | 0 |
| "tcDCS" AND "Stroke"[Mesh] | 0 |
| "transcutaneous spinal direct current stimulation" AND "Stroke"[Mesh] | 0 |
| "transspinal direct current stimulation" AND "Stroke"[Mesh] | 0 |
| "trans-spinal direct current stimulation" AND "Stroke"[Mesh] | 0 |
| "tsDCS" AND "Stroke"[Mesh] | 1 |
| "transcutaneous vagus nerve stimulation" AND "Stroke"[Mesh] | 1 |
| "taVNS" AND "Stroke"[Mesh] | 1 |
| "transcutaneous auricular vagus nerve stimulation" AND "Stroke"[Mesh] | 1 |
| "high-definition transcranial direct current stimulation" AND "Stroke"[Mesh] | 0 |
| "HD-tDCS" AND "Stroke"[Mesh] | 0 |
| "Theta Burst Stimulation" AND "Stroke"[Mesh] | 32 |
| "TBS" AND "Stroke"[Mesh] | 9 |
| "cerebellar repetitive transcranial magnetic stimulation" AND "Stroke"[Mesh] | 3 |
| "crTMS" AND "Stroke"[Mesh] | 0 |
| "Transcranial Magnetic Stimulation"[Mesh] AND "Brain ischemia"[Mesh] | 2 |
| "TMS" AND "Brain ischemia"[Mesh] | 4 |
| "repetitive Transcranial Magnetic Stimulation" AND "Brain ischemia"[Mesh] | 1 |
| "rTMS" AND "Brain ischemia"[Mesh] | 1 |
| "Noninvasive Brain Stimulation" AND "Brain ischemia"[Mesh] | 0 |
| "transcranial Direct Current Stimulation"[Mesh) AND "Brain ischemia"[Mesh] | 0 |
| "tDCS" AND "Brain ischemia"[Mesh] | 0 |
| "brain polarization" AND "Brain ischemia"[Mesh] | 0 |
| "transcranial alternating current stimulation" AND "Brain ischemia"[Mesh] | 0 |
| "tACS" AND "Brain ischemia"[Mesh] | 0 |
| "transcranial electrical stimulation" AND "Brain ischemia"[Mesh] | 0 |
| "tES" AND "Brain ischemia"[Mesh] | 0 |
| "transcranial random noise stimulation" AND "Brain ischemia"[Mesh] | 0 |
| "tRNS" AND "Brain ischemia"[Mesh] | 0 |
| "transcranial cerebellar direct current stimulation" AND "Brain ischemia"[Mesh] | 0 |
| "cerebellar direct current stimulation" AND "Brain ischemia"[Mesh] | 0 |
| "tcDCS" AND "Brain ischemia"[Mesh] | 0 |
| "transcutaneous spinal direct current stimulation" AND "Brain ischemia"[Mesh] | 0 |
| "transspinal direct current stimulation" AND "Brain ischemia"[Mesh] | 0 |
| "trans-spinal direct current stimulation" AND "Brain ischemia"[Mesh] | 0 |
| "tsDS" AND "Brain ischemia"[Mesh] | 0 |
| "transcutaneous vagus nerve stimulation" AND "Brain ischemia"[Mesh] | 0 |
| "taVNS" AND "Brain ischemia"[Mesh] | 0 |
| "transcutaneous auricular vagus nerve stimulation" AND "Brain ischemia"[Mesh] | 0 |
| "High-definition transcranial direct current stimulation" AND "Brain ischemia"[Mesh] | 0 |
| "HD-tDCS" AND "Brain ischemia"[Mesh] | 0 |
| "Theta Burst Stimulation" AND "Brain ischemia"[Mesh] | 0 |
| "TBS" AND "Brain ischemia"[Mesh] | 0 |
| "cerebellar repetitive transcranial magnetic stimulation" AND "Brain ischemia"[Mesh] | 0 |
| "crTMS" AND "Brain ischemia"[Mesh] | 0 |
| "Transcranial Magnetic Stimulation"[Mesh] AND "Intracranial hemorrhages"[Mesh] | 0 |
| "TMS" AND "Intracranial hemorrhages"[Mesh] | 0 |
| "repetitive Transcranial Magnetic Stimulation" AND "Intracranial hemorrhages"[Mesh] | 0 |
| "rTMS" AND "Intracranial hemorrhages"[Mesh] | 0 |
| "Noninvasive Brain Stimulation" AND "Intracranial hemorrhages"[Mesh] | 0 |
| "transcranial direct current stimulation" AND "Intracranial hemorrhages"[Mesh] | 0 |
| "tDCS" AND "Intracranial hemorrhages"[Mesh] | 0 |
| "brain polarization" AND "Intracranial hemorrhages"[Mesh] | 0 |
| "transcranial alternating current stimulation" AND "Intracranial hemorrhages"[Mesh] | 0 |
| "tACS" AND "Intracranial hemorrhages"[Mesh] | 0 |
| "transcranial electrical stimulation" AND "Intracranial hemorrhages"[Mesh] | 0 |
| "tES" AND "Intracranial hemorrhages"[Mesh] | 0 |
| "transcranial random noise stimulation" AND "Intracranial hemorrhages"[Mesh] | 0 |
| "tRNS" AND "Intracranial hemorrhages"[Mesh] | 0 |
| "transcranial cerebellar Direct Current Stimulation" AND "Intracranial hemorrhages"[Mesh] | 0 |
| "tcDCS" AND "Intracranial hemorrhages"[Mesh] | 0 |
| "transcutaneous spinal direct current stimulation" AND "Intracranial hemorrhages"[Mesh] | 0 |
| "transspinal direct current stimulation" AND "Intracranial hemorrhages"[Mesh] | 0 |
| "trans-spinal direct current stimulation" AND "Intracranial hemorrhages"[Mesh] | 0 |
| "tsDCS" AND "Intracranial hemorrhages"[Mesh] | 0 |
| "transcutaneous vagus nerve stimulation" AND "Intracranial hemorrhages"[Mesh] | 0 |
| "taVNS" AND "Intracranial hemorrhages"[Mesh] | 0 |
| "transcutaneous auricular vagus nerve stimulation" AND "Intracranial hemorrhages"[Mesh] | 0 |
| "High-definition transcranial Direct Current Stimulation" AND "Intracranial hemorrhages"[Mesh] | 0 |
| "HD-tDCS" AND "Intracranial hemorrhages"[Mesh] | 0 |
| "Theta Burst Stimulation" AND "Intracranial hemorrhages"[Mesh] | 0 |
| "TBS" AND "Intracranial hemorrhages"[Mesh] | 0 |
| "cerebellar repetitive Transcranial Magnetic Stimulation" AND "Intracranial hemorrhages"[Mesh] | 0 |
| "crTMS" AND "Intracranial hemorrhages"[Mesh] | 0 |
| "Non-invasive Brain Stimulation" AND "Stroke"[Mesh] | 43 |
| "NIBS" AND "Stroke"[Mesh] | 38 |
| "Non-invasive Brain Stimulation" AND "Brain ischemia"[Mesh] | 0 |
| "NIBS" AND "Brain ischemia"[Mesh] | 0 |
| "Non-invasive Brain Stimulation" AND "Intracranial hemorrhages"[Mesh] | 0 |
| "NIBS" AND "Intracranial hemorrhages"[Mesh] | 0 |
| **TOTAL** | **739** |
